# EPO Deficiency Upregulates GADD45b/p38 MAPK Axis, Mediating Schizophrenia‐Related Synaptic and Cognitive Impairments

**DOI:** 10.1002/advs.202406979

**Published:** 2024-10-28

**Authors:** Cuiping Guo, Wensheng Li, Yi Liu, Xiaoqing Tao, Yacoubou Abdoul Razak Mahaman, Jianzhi Wang, Rong Liu, Shusheng Li, Xiaochuan Wang

**Affiliations:** ^1^ Department of Pathophysiology School of Basic Medicine Key Laboratory of Education Ministry/Hubei Province of China for Neurological Disorders Tongji Medical College Huazhong University of Science and Technology Wuhan 430030 China; ^2^ Department of Emergency Medicine Tongji Hospital Tongji Medical College Huazhong University of Science and Technology Wuhan 430030 China; ^3^ Department of Critical Care Medicine Tongji Hospital Tongji Medical College Huazhong University of Science and Technology Wuhan 430030 China; ^4^ Hubei Key Laboratory of Cognitive and Affective Disorders Institute of Biomedical Sciences School of Medicine Jianghan University, China; ^5^ Co‐innovation Center of Neuroregeneration Nantong University Nantong 226001 China; ^6^ Shenzhen Huazhong University of Science and Technology Research Institute Shenzhen 518000 China

**Keywords:** cognitive impairments, erythropoietin, GADD45b, p38 MAPK, schizophrenia

## Abstract

Schizophrenia (SZ) is a chronic and severe mental illness associated with psychiatric symptoms, cognitive deficits, and social dysfunction. Current clinical interventions only limit relief of psychiatric symptoms and have minimal impact on cognitive impairments. Erythropoietin (EPO), known for its role in neurogenesis and synaptic plasticity, is significantly low in SZ patients. However, the role of EPO deficiency in SZ‐associated cognitive deficits remains unclear. In this study, we used the MK801‐induced SZ rat model to show that EPO levels were significantly decreased, correlating with cognitive impairments. EPO supplementation mitigated apoptosis, synaptic damage, and cognitive impairments caused by MK801. RNA‐sequencing and Western blot analysis revealed increased expression of growth arrest and DNA damage 45b (GADD45b) in MK801‐treated rats, reversed by EPO supplementation. Moreover, overexpression of GADD45b exacerbated neuronal loss and cognitive impairments in male Sprague‐Dawley rats, while downregulation of GADD45b rescued these SZ‐related pathologies. Notably, the benefits of EPO supplementation on SZ pathology were blocked by GADD45b overexpression. Inhibition of p38 MAPK, a GADD45b target, reduced MK801‐induced apoptosis and synaptic damage. These findings uncover a novel etiopathogenic mechanism of SZ‐related cognitive impairments, driven by EPO deficiency and the activation of the GADD45b/p38 MAPK axis.

## Graphical abstract

A novel etiopathogenic mechanism of SZ‐related cognitive impairments is uncovered, driven by EPO deficiency and the activation of the GADD45b/p38 MAPK axis. The results highlight potential therapeutic strategies for cognitive deficits in SZ.

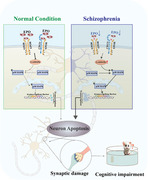

## 1 Introduction

Schizophrenia is a chronic and severe mental illness associated with psychosis, cognitive impairments, and social deficits.^[^ ^1^, ^2^, ^3^ ^]^ The incidence of mental illnesses has been increasing worldwide due to the acceleration of social rhythms and rising work pressures, with recent data suggesting an even higher lifetime risk.^[^ ^4^, ^5^ ^]^ Current clinical interventions primarily involve antipsychotic drugs, psychological counseling, and social support, but these only provide limited relief from psychotic symptoms and have minimal effect on cognitive disorders.^[^ ^6^, ^7^, ^8^ ^]^ To date, there is no optimal pharmacological intervention strategy, and the underlying mechanisms of cognitive dysfunction in schizophrenia remain unknown. Therefore, elucidating its molecular mechanisms is crucial for developing improved therapeutic strategies.

Increasing research indicates that abnormalities in dopamine and glutaminergic neurotransmission contribute to the development of mental illnesses, while key factors such as brain‐derived neurotrophic factor (BDNF) and NRG1 are implicated in the pathogenesis of cognitive disorders in schizophrenia.^[^ ^6^, ^9^, ^10^, ^11^ ^]^ However, the precise molecular mechanisms of these factors are yet to be elucidated. Recent studies report a significant reduction in erythropoietin levels in the brains of individuals with schizophrenia, suggesting a strong link between EPO deficiency and the onset of schizophrenia.^[^ ^12^, ^13^ ^]^ Additionally, EPO has been positively correlated with cognitive function.^[^ ^14^, ^15^ ^]^ Erythropoietin, initially identified as a hypoxia‐induced growth factor in mammalian kidneys, derives its name from its pivotal role in hematopoiesis, the process of blood cell formation.^[^ ^13^, ^16^ ^]^ Interestingly, high‐dose recombinant human EPO (rhuEPO), clinically used to treat anemia, has been demonstrated to possess neuroprotective and neuro‐regenerative potentials independent of its hematopoietic function, though the underlying mechanisms remain unclear. It has been established that EPO exerts its brain‐protective functions through the erythropoietin receptor (EPOR).^[^ ^17^, ^18^, ^19^, ^20^, ^21^ ^]^ Initially believed to exist only in early erythroid cells, EPOR has been shown by recent studies to play a significant role in the nervous system.^[^ ^22^, ^23^, ^24^ ^]^ Although the expression of EPO and EPOR in the normal postnatal brain is low,^[^ ^25^ ^]^ these proteins are distinctly expressed in areas such as the hippocampus and cortex.^[^ ^26^, ^27^ ^]^

The N‐methyl‐D‐aspartate receptor (NMDAR) channel blocker MK801, has been widely employed in SZ model to investigate schizophrenia‐related cognitive disorders.^[^ ^28^, ^29^, ^30^, ^31^ ^]^ Our previous study revealed cognitive impairments, synaptic damage, and neuronal apoptosis in this SZ animal model.^[^ ^30^ ^]^ The stress‐activated mitogen‐activated protein kinase (MAPK) plays a key role in balancing cell survival and death in extracellular and intracellular stress responses.^[^ ^32^ ^]^ Extensive studies on apoptosis have suggested that MAPK plays a crucial role as a transmitter of signals from the cell surface to the nucleus interior, integrating signals from various transmission points through transcriptional mechanisms, ultimately culminating in caspase activation.^[^ ^33^, ^34^ ^]^

The GADD45 family, consisting of GADD45a, GADD45b, and GADD45g, plays a crucial role in cellular responses to DNA damage and various stress signals, leading to growth arrest and apoptosis.^[^ ^35^ ^]^ Despite lacking enzymatic activity, GADD45 proteins exert their functions through interactions with p21, a cell cycle inhibitor, to promote G1 arrest.^[^ ^36^, ^37^, ^38^ ^]^ More importantly, GADD45 can bind to MTK1 (MEKK4) a critical regulator of stress‐activated MAPK signaling.^[^ ^39^ ^]^ GADD45/MTK1 binding disrupts the auto‐inhibitory domain of MTK1, promoting its activation and the downstream induction of c‐Jun N‐terminal kinase (JNK) and p38, two members of the stress‐activated MAPK pathway, whose persistent activation promotes apoptosis.^[^ ^40^, ^41^ ^]^

In this report, we demonstrated that EPO deficiency led to SZ‐related cognitive impairments. In addition, supplementation of EPO improved synaptic and cognitive impairments by reducing GADD45b in SZ. Blockage of p38 MAPK attenuated apoptosis and synaptic damage. Hence, our results support a new insight into the mechanisms underlying cognitive impairments in SZ and provide a basis for potential therapeutic strategies.

## 2 Results

### 2.1 EPO Deficiency was Strongly Linked to Schizophrenia‐Related Cognitive Impairments

**Figure 1:**
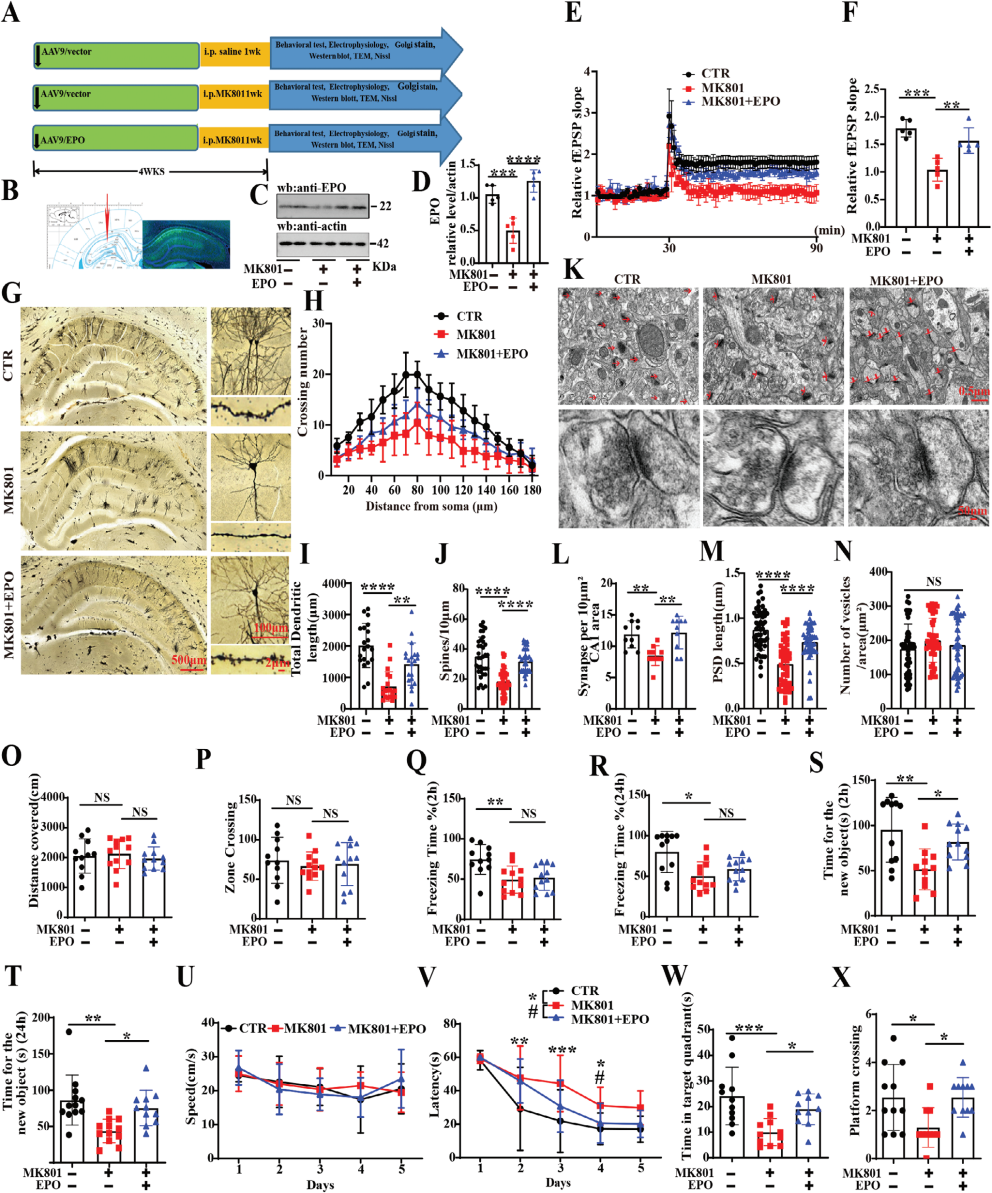
EPO deficiency was strongly linked to schizophrenia‐related cognitive impairments. (A) Experimental design schematic. Thirty‐six healthy SD rats were randomly divided into three groups. In the control, 12 rats were injected with AAV9/CTR virus in the hippocampal CA1 region. In the Mod group, 12 rats were daily injected intraperitoneally with MK801 (0.3 mg kg^−1^) for 1 week. In the preventive group, 12 rats were injected with AAV9/EPO virus in the hippocampal CA1 region and with simultaneous daily intraperitoneal injection of MK801 (0.3 mg kg^−1^) for the last 1 week. Following the treatment, behavioral, electrophysiological, and biochemical tests were performed. (B) AAV9/EPO was injected into the hippocampal CA1 region of the SD rats, and GFP expression was observed (green) 4 weeks after injection. (C) Brain tissues (hippocampus) from the three groups were homogenized and EPO protein levels were detected by immunoblotting. Actin was used as a loading control. (D) Quantitative analysis of the EPO, (*n* = 5). (E) Hippocampal CA3‐CA1 LTP and its quantification (F) were recorded by using the MED64 system, (*n* = 5). (G) Representative dendrite from Golgi‐impregnated hippocampus neurons, Scale bar: 500 µm. Sholl analysis, (*n* = 15), (H) quantitative analyses of dendritic length (I), (Scale bar: 100 µm, *n* = 20), and averaged spine density (mean spine number per 10‐µm dendrite segment) (J), (Scale bar: 2 µm, *n* = 35). (K) Representative electron micrographs of the synaptic structures, arrows indicate synapses. Quantitative analysis of the synaptic density (Scale bar: 0.5 µm, n = 10 from 3 rats per group) (L). The length of postsynaptic density (M) and the release of presynaptic vesicles (N), (Scale bar: 50 nm, *n* = 50). (O,P) The open field test showed the total distance covered (O) and the zone crossing (P), (*n* = 10–12). (Q,R) Fear conditioning test was used to measure the contextual memory: freezing duration was measured during the 3‐min memory test at 2 h (Q) or 24 h (R) after conditioning, (*n* = 10–12). (S,T) The test showed the measured recognition time of the new object in 2 h (S) and 24 h (T) (*n* = 10–12). (U–X) The Morris water maze test: the swimming speed (U) and the latency to find the hidden platform (V) from day 1 to day 5, the spatial memory was tested on the sixth day by removing the platform, and the time spent in the target quadrant (W) and the number of crossing the position of the target platform (X) were measured, (*n* = 10–12). Data are presented as Mean ± SD. ^*^ *p* < 0.05, #*p* < 0.05, ^**^ *p* < 0.01, ^***^ *p* < 0.001, ^****^ *p* < 0.0001, versus Mod group. Statistical details were provided in Table S2 (Supporting Information).

Our previous study revealed that cognitive impairments and synaptic dysfunction were accompanying symptoms of schizophrenia.^[^ ^30^ ^]^ In this study, the NMDA receptor channel blocker MK801 was used to establish a schizophrenia animal model. Meanwhile, EPO levels in the brains of individuals with schizophrenia were markedly low.^[^ ^12^, ^13^ ^]^ We also detected EPO levels were reduced in the hippocampus (**Figure** **1**C,D) and cortex (Figure S1A,B, Supporting Information) in MK801 rats. To explore whether EPO deficiency is linked to the occurrence of cognitive impairments in SZ, 8‐week‐old SD rats were randomly divided into three groups (control, MK801 model, and MK801+EPO preventive groups), behavioral, electrophysiological, and biochemical tests were performed (Figure 1A). GFP expression in the hippocampal CA1 region was confirmed by fluorescence microscopy 4 weeks after injection (Figure 1B). Western blotting results showed that the levels of EPO protein in SZ rats were significantly reduced, while adeno‐associated virus 9(AAV9)/EPO infection increased the levels of EPO protein to a normal level (Figure 1C,D). Meanwhile, EPOR levels were increased by supplementation with EPO in MK801+EPO rats (Figure S2A,B, Supporting Information). We wondered whether EPO deficiency mediated schizophrenia‐related synaptic dysfunction. Thus, we carried out electrophysiology experiments and found that SZ animals exhibit a decrease in the slope of field excitatory postsynaptic potential (fEPSP) after high‐frequency stimulation (HFS) compared to the control group (Figure 1E). Notably, we observed that the fEPSP in the MK801+EPO group was higher than that in the Mod SZ group (Figure 1F). In addition, we examined the dendritic architecture of hippocampal CA1 neurons (Figure 1G). The results from Golgi staining showed that EPO resulted in an obvious increase in the dendritic complexity at all points farther than 80 µm from the cell body compared to the Mod group (Figure 1H), as well as a significant increase in total dendritic length (Figure 1I) and the dendritic spine density (Figure 1J). Transmission electron microscopy showed that compared to the Mod group, the number of synapses per area of CA1 (Figure 1K,L) and the length of the postsynaptic density(PSD) of a single synaptic structure (Figure 1M) were significantly increased to normal in the MK801+EPO group, while the number of synaptic vesicles had no significant difference among each group (Figure 1N). These results implied that supplementation of EPO blocks MK801‐induced SZ‐like synaptic damage.

Next, we again performed a series of behavioral tests. The open field test was used to assess the autonomous motor ability and the results showed no significant difference in the total distance covered (Figure 1O) and the number of times in the zone crossing (Figure 1P) among groups. The Fear condition test, which explores contextual learning and memory ability, showed that the freezing duration in the Mod group was significantly reduced at 2 h (Figure 1Q) and 24 h (Figure 1R), while the MK801+EPO rats showed no significant difference at these time points compared to the Mod group. The novel object recognition (NOR) test was used to assess short‐term memory and the results demonstrated a significant increase in the curiosity for exploring novel stimuli in the MK801+EPO group, indicated by the increased time spent discovering a new object at 2 h (Figure 1S) and 24 h (Figure 1T) compared to the Mod group. Finally, we tested memory and learning abilities using the Morris water maze and observed that the MK801+EPO rats showed a significantly decreased latency to find the hidden platform (Figure 1V) compared to the Mod group, with no significant difference in swimming speed among the three groups (Figure 1U). On day 6, spatial memory was tested by removing the platform. A remarkable increase in the time spent in the target quadrant (Figure 1W), without an increase in the number of platform crossing (Figure 1X), was observed in the MK801+EPO group compared to the Mod group. Taken together, these behavioral tests suggest that EPO deficiency may be linked to SZ‐related cognitive impairments.

### 2.2 EPO Deficiency Induced a Significant Expression of GADD45b

**Figure 2:**
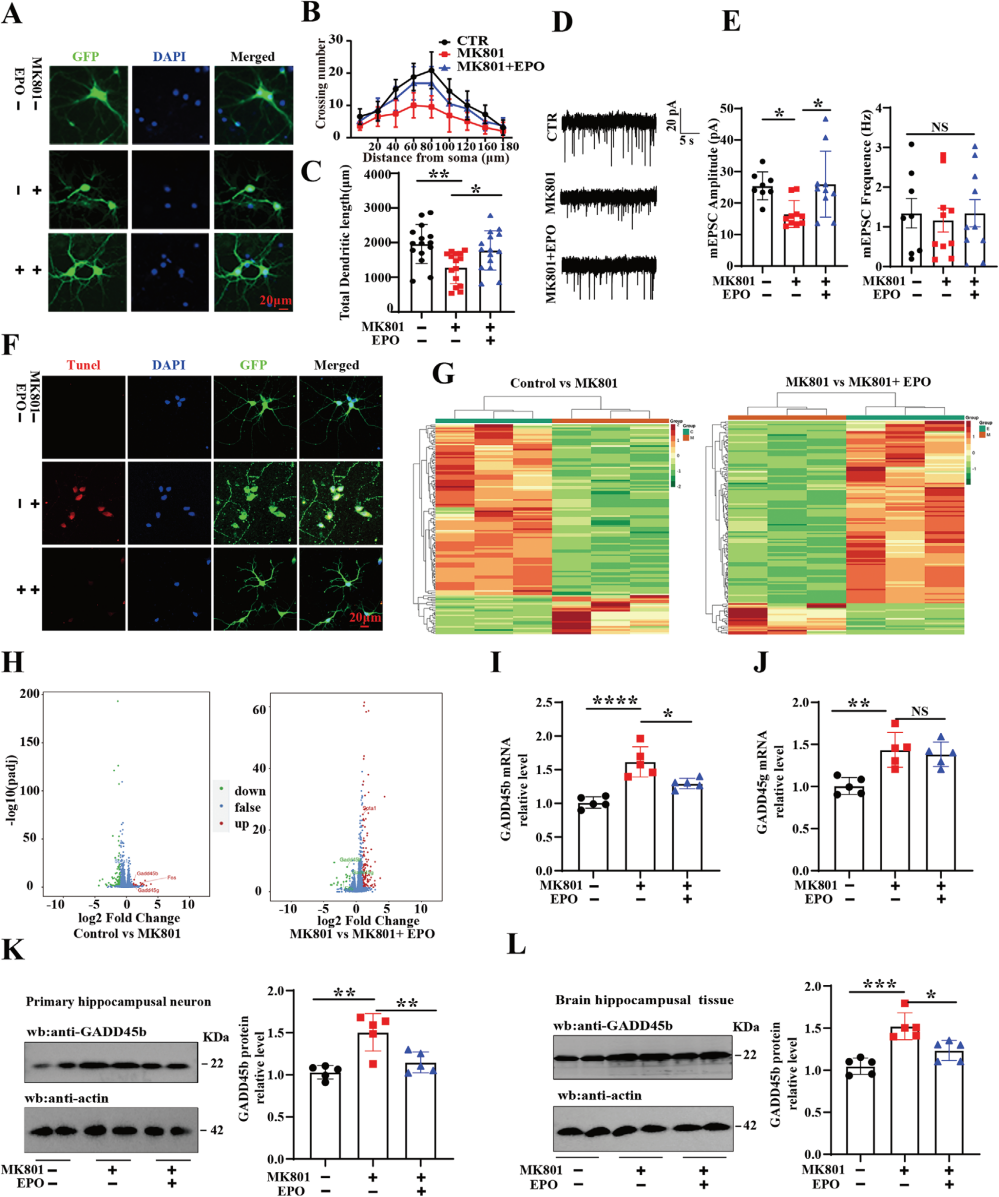
EPO deficiency induced a significant expression of GADD45b. (A–C) Rats’ primary hippocampal neurons were treated with AAV9/EPO virus and MK801 on the ninth day. Representative images after treatment (A) (Scale bar: 20 µm), Sholl analysis (B), quantitative analyses of dendritic length (C), (*n* = 15). (D,E) Rat hippocampal neurons were subjected to whole‐cell patch‐clamp recording of mEPSCs (D), amplitude and frequency (E) of mEPSCs measured, (*n* = 8–10). (F) Representative images of TUNEL staining were shown, (Scale bar: 20 µm). (G) The clustering heat map showed the expression of differential genes. (H) GADD45b/GADD45 g mRNA expression was measured by RNA sequencing. The mRNA levels of GADD45b (I) and GADD45 g (J) were measured by QPCR, (*n* = 5). (K) Rats’ primary hippocampal neurons were homogenized, and GADD45b protein levels were detected by immunoblotting. Actin was used as a loading control (*n* = 5). (L) Brain tissues (hippocampus CA1 region) were homogenized, and GADD45b protein levels were detected by immunoblotting. Actin was used as a loading control, (*n* = 5). Data are presented as Mean ± SD. ^*^ *p* < 0.05, ^**^ *p* < 0.01, ^***^ *p* < 0.001, ^****^ *p* < 0.0001, versus Mod group. Statistical details were provided in Table S2 (Supporting Information).

The SZ rats exhibited cognitive impairments and synaptic damage, indicating that modulating EPO may be a therapeutic option for SZ‐related cognitive impairments. Primary hippocampal neurons were transduced with AAV9/EPO virus and treated with MK801 on the ninth day, and AAV9/GFP was used to examine dendritic morphology (**Figure** **2**A). Interestingly, supplementation of EPO resulted in an obvious increase in the dendritic complexity at all points farther than 80 µm from the cell body and the total dendritic length when compared with MK801 treated neurons (Figure 2B,C). Whole‐cell patch‐clamp was used to record miniature Excitatory Postsynaptic Current (mEPSC) in primary hippocampal neurons (Figure 2D) and the results showed that EPO significantly improved the amplitude, but no significant difference in the frequency of mEPSC (Figure 2D,E).

Our previous study revealed that MK801 induced neuronal apoptosis.^[^ ^30^ ^]^ In this study, TUNEL staining demonstrated that EPO rescued cell apoptosis induced by MK801 (Figure 2F). To further explore the possible molecular mechanism of how supplementation of EPO improves synaptic damage in the hippocampus, we performed RNA sequencing and the results revealed differential expression of genes in the three groups as shown by the clustering heat map (Figure 2G). The data revealed a noticeable decrease in the GADD45 gene, a key component of the apoptotic pathway, observed in the MK801+EPO group compared to the Mod group (Figure 2H), suggesting an inhibition of the apoptosis pathway. The GADD45 family, consisting of GADD45a, GADD45b, and GADD45g, is usually induced by DNA damage and other stress signals associated with growth arrest and apoptosis. Consistently, qPCR analysis showed an upregulation of both GADD45b (Figure 2I) and GADD45g (Figure 2J) in the Mod group, and a downregulation of GADD45b following supplementation of EPO (Figure 2I), with no significant difference in GADD45g (Figure 2J). Similarly, Western blotting from primary hippocampal neurons (Figure 2K) as well as an in vivo experiment (Figure 2L), showed down‐regulated GADD45b levels to normal after supplementation of EPO compared to the Mod group. Together, these data suggest that EPO deficiency may be involved in SZ‐related cognitive impairments by activating the GADD45b‐related apoptosis pathway.

### 2.3 Overexpression of GADD45b Resulted in Synaptic Damage and Learning and Memory Impairments

**Figure 3:**
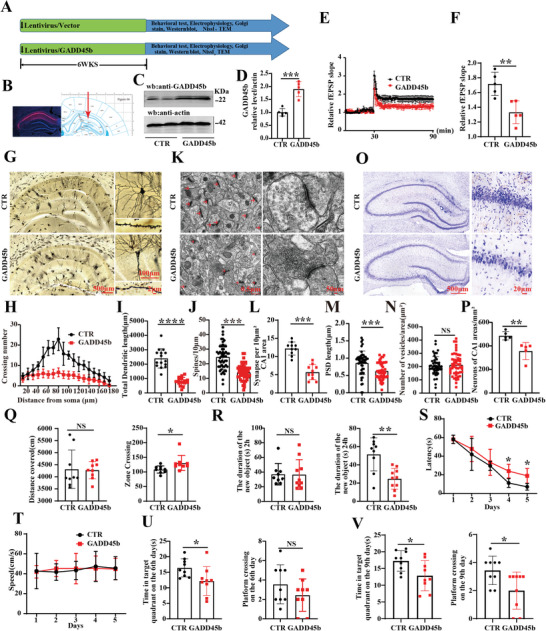
Overexpression of GADD45b resulted in synaptic damage and learning and memory impairments. (A)Experimental design: 20 healthy SD rats were randomly divided into two groups. In the control group, ten rats were injected with LV/vector virus in the hippocampal CA1 region, while in the GADD45b group, ten rats were daily injected with LV/GADD45b virus in the hippocampal CA1 region. Following the treatment, behavioral, electrophysiological, and biochemical tests were performed. (B) LV/GADD45b Injection: LV/GADD45b was injected into the hippocampal CA1 region of the SD rats, and mCherry expression (red) was observed 6 weeks after injection. (C) Brain tissues (hippocampus CA1) were homogenized, and GADD45b protein levels were detected by immunoblotting. Actin was used as a loading control. (D) Quantitative analysis of GADD45b expression, (*n* = 5). (E) Hippocampal CA3‐CA1 LTP was recorded using the MED64 system. (F) The normalized CA3‐CA1 fEPSP mean slope recorded from the CA1 dendritic region in hippocampal slices was analyzed, (*n* = 5). (G) The representative dendrite of Golgi impregnated hippocampal neurons, (Scale bar: 500 µm). Sholl analysis (Scale bar: 100 µm, *n* = 15) (H), quantitative analyses of dendritic length (Scale bar: 100 µm, *n* = 15) (I), and averaged spine density (mean spine number per 10‐µm dendrite segment) (Scale bar: 2 µm, *n* = 50) (J). (K) Representative electron micrographs, (scale bar = 0.5 µm), with arrows indicating the synapses. (L) Quantitative analysis of synaptic density, (Scale bar: 0.5 µm, *n* = 10 from three rats per group). (M) Quantitative analysis was conducted to measure the length of postsynaptic densities (Scale bar: 50 nm, *n* = 50). (N) Quantitative analysis was performed to assess the release of presynaptic vesicles (Scale bar: 50 nm, *n* = 50). (O) Representative Nissl staining of the hippocampus, (Scale bar: 500 µm). (P) The quantitative analysis of the number of neurons in the hippocampal CA1 regions was conducted, (*n* = 5). (Q) The open field test measured the total distance covered and zone crossing in the two groups (*n* = 9‐10). (R) The NO test measured the recognition time of the new object at 2 h and 24 h (*n* = 9–10). (S–V) Morris Water Maze Test: latency to find the hidden platform (S) and Swimming speed (T) from day 1 to day 5 were recorded. Spatial memory was assessed by measuring the time spent in the target quadrant (U) and the number of target platform crossing (V) on the sixth and ninth days, (*n* = 9‐10). Data are presented as Mean ± SD. ^*^ *p* < 0.05, ^**^ *p* < 0.01, ^***^ *p* < 0.001, ^****^ *p* < 0.0001, versus GADD45b group. Statistical details were provided in Table S2 (Supporting Information).

To further determine the effect of GADD45b on cognitive function, SD rats were randomly divided into two groups of ten rats (GADD45b group and control group). We performed bilateral intra‐hippocampal CA1 injection of GADD45b lentivirus(LV) and vector served as control (**Figure** **3**A). mCherry expression in the hippocampal CA1 was confirmed by fluorescence microscopy 6 weeks after injection (Figure 3B), while Western blotting data indicated that GADD45b protein levels were increased in GADD45b group (Figure 3C,D). The electrophysiology experiments showed that GADD45b reduced the fEPSP after high‐frequency stimulation compared to the control group (Figure 3E,F). We further examined the dendritic architecture of the hippocampal neurons using Golgi staining, which showed that GADD45b resulted in an obvious decrease in the dendritic complexity at all points farther than 80 µm from the cell body compared to the control group (Figure 3G,H). Moreover, a significant decrease in the total dendritic length (Figure 3I) and dendritic spine density (Figure 3J) was also observed. Consistently, transmission electron microscopy results showed a significant decrease in the number of synapses per area of CA1 (Figure 3K,L) and the length of the postsynaptic density (Figure 3M) with no significant difference in the number of synaptic vesicles (Figure 3N) following GADD45b overexpression. These results suggest that an increase in GADD45b is associated with synaptic dysfunction. GADD45b is typically induced by apoptosis, and in line with this, the Nissl staining showed that GADD45b induced neuronal loss in the hippocampal CA1 region (Figure 3O,P), with no significant difference observed in the hippocampal CA3 and dentate gyrus(DG) regions (Figure S3A–C, Supporting Information). We again performed additional behavioral tests. Open‐field test data showed no significant difference in the total distance covered, but the number of times in the zone crossing was increased in the GADD45b group (Figure 3Q). The results of the NOR showed a decreased curiosity for exploring new things, indicated by the decreased time spent discovering a new object at 24 h only, but not at 2 h (Figure 3R) in the GADD45b group compared to the control group. Finally, the data from MWM support that the GADD45b rats exhibit a significantly increased latency to find the hidden platform (Figure 3S) compared to the control group, with no significant change in the swimming speed (Figure 3T) between the two groups. On the sixth day, the data showed a significant decrease in the time spent in the target quadrant for the GADD45b group compared to the control group, with no increase in the number of target platform crossing (Figure 3U). However, on the ninth day, there was a noticeable decrease in both the time spent in the target quadrant and the number of target platform crossing in the GADD45b group (Figure 3V). These findings collectively indicate that GADD45b may contribute to learning and memory impairments, as well as synaptic damage.

### 2.4 Down‐Regulation of GADD45b Improved MK801‐Induced Synaptic and Cognitive Deficits

**Figure 4:**
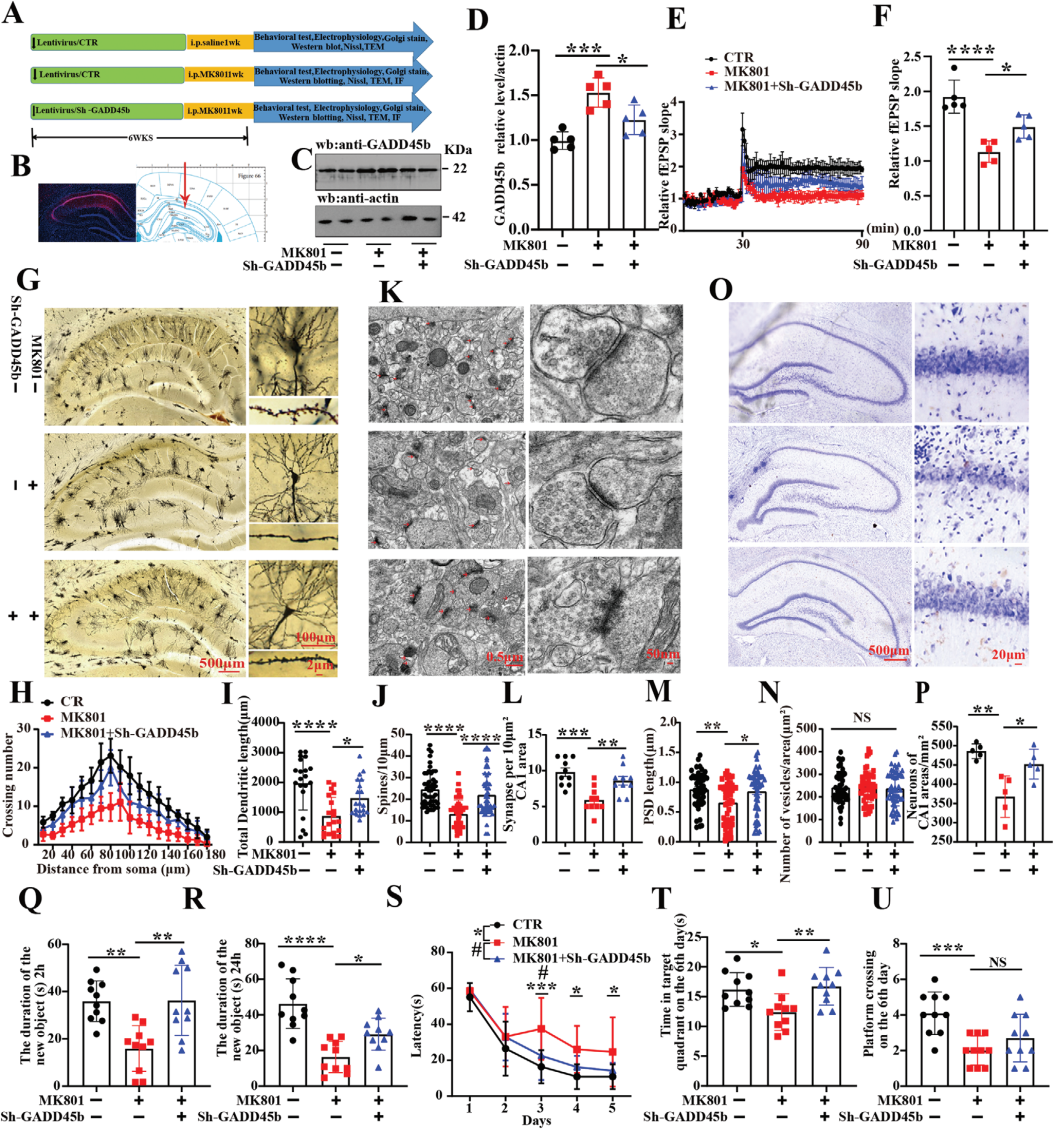
Down‐regulation of GADD45b improved MK801‐induced synaptic and cognitive deficits. (A) Experimental design schematic. Thirty healthy SD rats were randomly divided into three groups. In the control group, ten rats were injected with LV/vector virus in the hippocampal CA1 region. In the model (Mod) group, ten rats were injected intraperitoneally with MK801 (0.3 mg kg^−1^) daily for 1 week. In the Pre group, ten rats were injected with LV/Sh‐GADD45b virus in the hippocampal CA1 region and received daily intraperitoneal injections of MK801 (0.3 mg kg^−1^) for 1 week. Following the treatment, behavioral, electrophysiological, and biochemical tests were performed. (B) mCherry expression was observed (red) 6 weeks after injection. (C) Brain tissues (hippocampus CA1 region) were homogenized, and GADD45b protein levels were detected by immunoblotting. Actin was used as a loading control. (D) Quantitative analysis of the GADD45b, (*n* = 5). (E) Hippocampal CA3‐CA1 Long‐Term Potentiation was recorded using the MED64 system. (F) The normalized mean slope of CA3‐CA1 fEPSP was recorded from the CA1 dendritic region in hippocampal slices (*n* = 5). (G) Representative dendrite from Golgi impregnated hippocampal neurons, (Scale bar: 500 µm). Sholl analysis, (Scale bar: 100 µm, *n* = 15) (H), quantitative analyses of dendritic length (Scale bar: 100 µm, *n* = 20) (I), and averaged spine density (mean spine number per 10‐µm dendrite segment) (Scale bar: 2 µm, *n* = 50) (J). (K) Representative electron micrographs of synaptic structures, with arrows indicating the synapses. Quantitative analysis of synaptic density (Scale bar: 0.5 µm, *n* = 10 from three rats per group) (L). Measurement of postsynaptic density length (M) and quantification of presynaptic vesicle release (N), (Scale bar: 50 nm, *n* = 50). (O) Representative Nissl staining of the hippocampus. (P) Quantitative analysis of the number of hippocampal CA 1 region (Scale bar: 500 µm, *n* = 5). (Q,R) Novel object recognition test showed the measured recognition at 2 h (Q) and 24 h (R), (*n* = 10). (S–U) The Morris water maze test evaluated the latency to locate the hidden platform from day 1 to day 5 (S). Spatial memory was assessed by removing the platform on the sixth day, with measurements of the time spent in the target quadrant (T) and the number of crossing over the position of the target platform (U), (*n* = 10). Data are presented as Mean ± SD. ^*^ *p* < 0.05, #*p* < 0.05, ^**^ *p* < 0.01, ^***^ *p* < 0.001, ^****^ *p* < 0.0001, versus MK801 group. Statistical details were provided in Table S2 (Supporting Information).

To investigate whether GADD45b plays an important role in SZ‐related cognitive impairments, we conducted bilateral intra‐hippocampal CA1 injections of either vector or Sh‐GADD45b virus in 8‐week‐old SD rats. SD rats were randomly allocated into three groups of ten animals each (**Figure** **4**A). mCherry expression in the hippocampal CA1 region was confirmed by fluorescence microscopy 6 weeks after injection (Figure 4B) and the results showed decreased GADD45b protein levels in the MK801+Sh‐GADD45b group (Figure 4C,D). Electrophysiology experiments showed that GADD45b knockdown improved the slope of field excitatory postsynaptic potential after high‐frequency stimulation compared to the Mod group (Figure 4E,F). In addition, we further examined the dendrites of hippocampal neurons (Figure 4G). Golgi staining showed that GADD45b knockdown resulted in an obvious increase in the dendritic complexity at all points farther than 80 µm from the cell body (Figure 4H), as well as a significant increase in the total dendritic length (Figure 4I) and dendritic spine density (Figure 4J). Transmission electron microscopy was employed to observe synaptic structures (Figure 4K). The number of synapses per area of CA1 (Figure 4L) and the length of the postsynaptic density in a single synaptic structure (Figure 4M) were significantly increased in the MK801+Sh‐GADD45b group than in the Mod group. No significant difference was observed in the number of synaptic vesicles (Figure 4N) among the three groups. Nissl staining revealed that compared to the Mod group, downregulating GADD45b mitigated the reduction in the neurons in the CA1 region (Figure 4O,P). Subsequently, we conducted behavioral tests to corroborate these findings. The results of open field showed no significant difference in the total distance covered and the number of times in the zone crossing (Figure S4A,B, Supporting Information).The results of the NOR showed that the curiosity for exploring new things was significantly increased in the rats of the MK801+Sh‐GADD45b group, as indicated by the increased time spent discovering a new object at 2 h (Figure 4Q) and 24 h (Figure 4R) compared to the Mod group. Additionally, MWM results demonstrated a significantly shortened latency in locating the hidden platform in the MK801+Sh‐GADD45b rats compared to the Mod group (Figure 4S). On day 6, spatial memory was assessed by removing the platform. The data revealed a markedly increased time spent in the target quadrant for the MK801+Sh‐GADD45b group (Figure 4T), although no increase in the number of crossing over the target platform position was observed compared to the Mod group (Figure 4U). Taken together, these findings strongly suggest that increased GADD45b is involved in SZ‐related synaptic dysfunction and cognitive impairments.

### 2.5 Overexpression of GADD45b Blocked the Protective Effects of EPO in MK801‐Induced SZ

**Figure 5:**
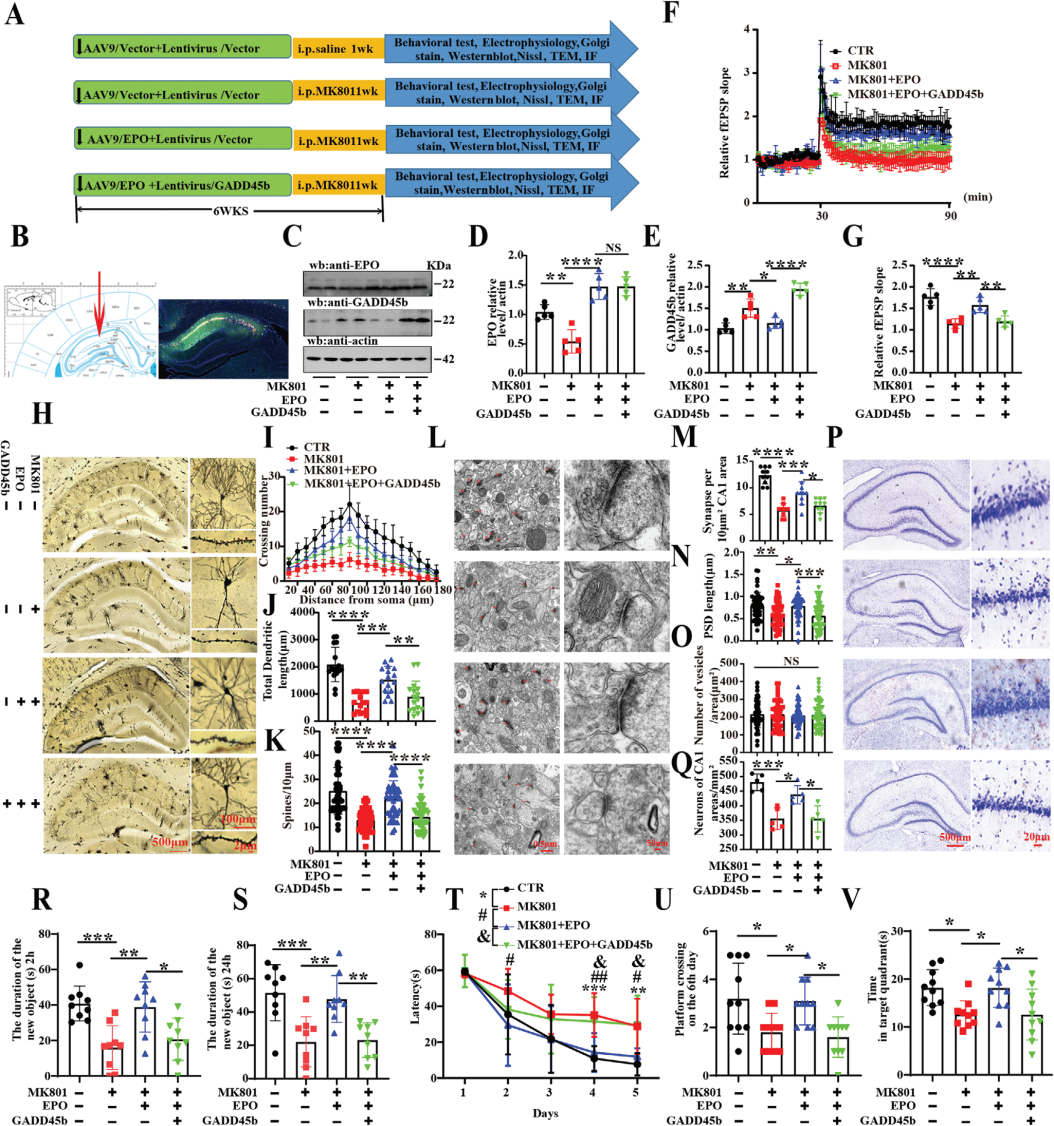
Overexpression of GADD45b blocked the protective effects of EPO in MK801‐induced SZ. (A) Experimental Design: 40 healthy SD rats were randomly divided into four groups. In the control group, ten rats received injections of vector virus in the hippocampal CA1 region. In the model (Mod) group, ten rats received daily intraperitoneal injections of MK801 (0.3 mg kg^−1^) for 1 week. In the preventive group ten rats were injected with AAV9/EPO virus in the hippocampal CA1 and received daily intraperitoneal injections of MK801 (0.3 mg kg^−1^) for 1 week. The remaining ten rats were injected with LV/GADD45b and AAV/EPO virus in the hippocampal CA1 and received daily intraperitoneal injections of MK801 (0.3 mg kg^−1^) for the 1 week and constitute the MK801+EPO+GADD45b group. (B) Fluorescence microscopy confirmed mCherry and GFP expression in the hippocampal CA1 6 weeks after injection. (C) Brain tissues (hippocampus CA1) were homogenized, and EPO and GADD45b protein levels were detected by immunoblotting. (D,E) Quantitative analysis of EPO and GADD45b expression was conducted, (*n* = 5). (F) Hippocampal CA3‐CA1 LTP recordings were performed using the MED64 system. (G) Normalized CA3‐CA1 fEPSP mean slope was recorded from the CA1 dendritic region in hippocampal slices, (*n* = 5). (H–K) Representative dendrites of neurons from the Golgi‐impregnated hippocampus (Scale bar: 500 µm) were analyzed using Sholl analysis (Scale bar: 100 µm, *n* = 16) (H,I) Quantitative analyses of dendritic length (Scale bar:100 µm, *n* = 16) (J) and averaged spine density (Scale bar:2 µm, *n* = 50) (K) were conducted. (L) Representative electron microscopy images of synaptic structures were obtained, with arrows indicating synapses. Quantitative analysis of synaptic density (Scale bar: 0.5 µm, *n* = 10) (M), length of postsynaptic densities (*n*), and presynaptic vesicle release (O) were performed, (Scale bar: 50 nm, *n* = 50). (P) Nissl staining of the hippocampus was shown. (Q) Quantitative analysis of the number of neurons in the hippocampal CA1 regions, (Scale bar: 500 µm, *n* = 5). (R, S) The recognition time of the new object was measured at 2 h (R) and 24 h (S), (*n* = 9‐10). (T‐V) Morris Water Maze Test: Latency to find the hidden platform from day 1 to day 5 (T) was recorded. Spatial memory was assessed by measuring the time spent in the target quadrant (U) and the number of target platform crossing (V) on the 6th day, (*n* = 9‐10). Data are presented as Mean ± SD. ^*^ *p* < 0.05, &*p* < 0.05, ^**^ *p* < 0.01, ^***^ *p* < 0.001, ^****^ *p* < 0.0001, #*p* < 0.05, ##*p* < 0.01, versus MK801 or MK801+EPO group. Statistical details were provided in Table S2 (Supporting Information).

We have shown that EPO could improve SZ‐related cognitive dysfunction, which may be mediated by inhibiting the expression of GADD45b. To further confirm this speculation, SD rats were randomly divided into four groups (control, MK801, MK801+EPO, MK801+EPO+GADD45b group) and treated as shown in (**Figure** **5**A). mCherry and GFP expression in the hippocampal CA1 were confirmed by fluorescence microscopy 6 weeks after injection (Figure 5B). Western blotting results showed increased GADD45b protein levels in the MK801+EPO+GADD45b group, while GADD45b overexpression did not affect the EPO levels in the MK801+EPO+GADD45b group (Figure 5C–E). We carried out electrophysiology experiments and found that in the MK801+EPO+GADD45b group, the fEPSP was lower than in the Pre group (Figure 5F,G). In addition, we examined the dendrites of hippocampal neurons (Figure 5H). The data showed that GADD45b in the MK801+EPO+GADD45b group resulted in an obvious decrease in the dendritic complexity at all points farther than 80 µm from the cell body when compared with MK801+EPO (Pre) group (Figure 5I), as well as a significant decrease in the total dendritic length (Figure 5J) and dendritic spine density (Figure 5K). The number of synapses per area of CA1 (Figure 5L,M) and the length of the postsynaptic density (Figure 5N) was also significantly decreased in the MK801+EPO+GADD45b group than in the MK801+EPO group, with no significant difference in the number of synaptic vesicles (Figure 5O). Moreover, Nissl staining showed that GADD45b induced neuronal loss in the CA1 region in the MK801+EPO+GADD45b group compared to the MK801+EPO group (Figure 5P,Q), with no significant difference in the hippocampal CA3 and DG regions (Figure S5A–C, Supporting Information). To further corroborate our findings, primary hippocampal neurons were transduced with either AAV9/EPO, LV/GADD45b virus, or untreated, and subsequently treated with MK801 on the ninth day. GFP and mCherry were used to examine dendritic morphology (Figure S6A, Supporting Information). GADD45b resulted in an obvious decrease in the dendritic complexity at all points farther than 80 µm from the cell body when compared with MK801+EPO neurons (Figure S6B,C, Supporting Information), suggesting that GADD45b suppresses EPO's protective effects against MK801‐induced synaptic dysfunction. Further, the results of the open field showed no significant difference in the total distance covered and the number of times in the zone crossing (Figure S7A,B, Supporting Information). While the results of the NOR revealed a significantly decreased curiosity for exploring new things in the MK801+EPO+GADD45b group, as indicated by the decreased time spent discovering a new object both within 2 h (Figure 5R) and 24 h (Figure 5S) compared to the MK801+EPO group. Furthermore, the MWM results indicate a significantly increased latency to find the hidden platform in the MK801+EPO+GADD45b compared to the MK801+EPO group (Figure 5T). On day 6, spatial memory test results revealed a remarkable decrease in both the number of target platform crossing (Figure 5U) and the time spent in the target quadrant (Figure 5V) in the MK801+EPO+GADD45b group compared to the MK801+EPO group. These results together imply that GADD45b blocks the protective effects of EPO in MK801‐induced SZ‐like synaptic and cognitive dysfunction.

### 2.6 EPO Deficiency Upregulated GADD45b/p38 MAPK Axis Mediating Apoptosis and Synaptic Impairments in SZ Rats

**Figure 6:**
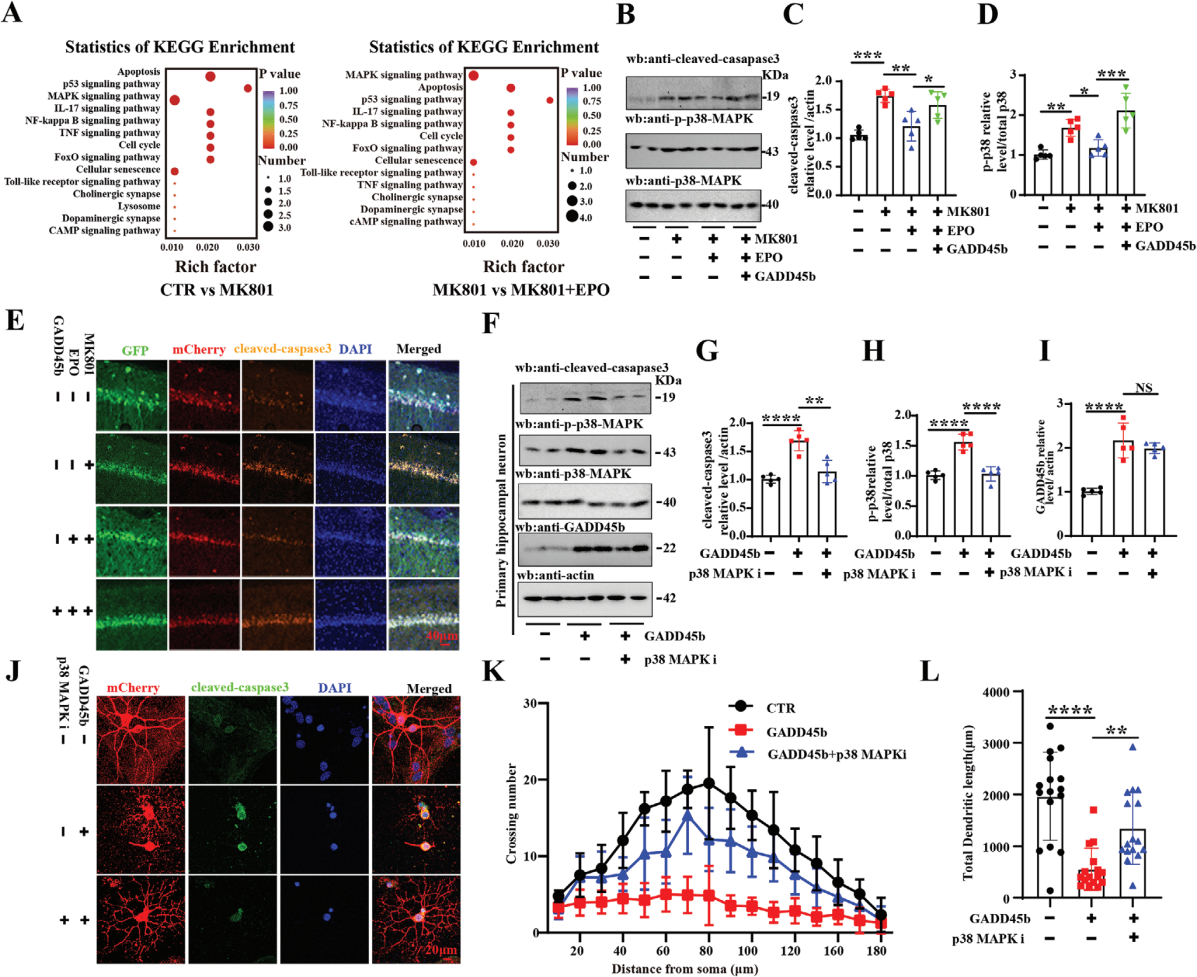
EPO deficiency upregulated GADD45b/p38 MAPK axis mediating apoptosis and synaptic impairments in SZ rats. (A) KEGG analysis of the differential RNA expression was shown. (B) Brain tissues (hippocampus CA1) were homogenized and cleaved‐caspase3 and p‐38 MAPK/p38 MAPK protein levels were detected by immunoblotting. Actin was used as a loading control. (C,D) Quantitative analysis of the cleaved‐caspase3 (C) and p‐P38 MAPK/p38 MAPK (D), (*n* = 5). (E) The cleaved‐caspase3 was measured by immunofluorescence in the four groups, (Scale bar: 40 µm). (F) Primary hippocampal neurons were homogenized, and cleaved‐caspase3, GADD45b, p‐p38 MAPK/p38 MAPK protein levels were detected by immunoblotting. Actin was used as a loading control. Quantitative analysis of the cleaved‐caspase3 (G), p‐p38 MAPK/p38 MAPK (H) and GADD45b (I) (*n* = 5). (J) Primary hippocampal neurons were transduced with LV/GADD45b virus, treated with p38 MAPK inhibitor on the ninth day, and the cleaved‐caspase3 was measured by immunofluorescence. Sholl analysis (K) and quantitative analyses of dendritic length (L), (Scale bar: 20 µm, *n* = 16 hippocampal neurons). Data are presented as Mean ± SD. ^*^ *p* < 0.05, ^**^ *p* < 0.01, ^***^ *p* < 0.001, ^****^ *p* < 0.0001, versus MK801or GADD45b or MK801+EPO group. Statistical details were provided in Table S2 (Supporting Information).

Our findings may suggest, in part, that an increase in GADD45b mediates EPO deficiency‐associated SZ. To uncover the underlying mechanism of our hypothesis, we further made the KEGG analysis of RNA sequencing (**Figure** **6**A) and found that EPO downregulates the MK801‐induced p38 MAPK apoptosis pathway. Actually, GADD45b lacks enzymatic activity and thus mediates its effects via interactions with partner proteins. For instance, GADD45/MTK1 binding promotes the activation of MTK1, and subsequently induces the downstream p38 MAPK pathway, whose persistent activation promotes apoptosis.^[^ ^40^, ^41^ ^]^ Therefore, we detected p38 MAPK pathway, apoptosis, and synaptic function in SZ rats. Western blotting showed that the levels of cleaved‐caspase3 and phosphorylated p38 MAPK were significantly increased in the SZ group which was blocked by EPO treatment (Figure 6B–D). Moreover, overexpression of GADD45b inhibited the protective effect of EPO on p38 MAPK‐related apoptosis in SZ rats (Figure 6B–D), while p‐JNK/JNK levels were no significant difference at the supplementation with EPO in SZ model (Figure S8A,B, Supporting Information). Immunofluorescence results showed that GADD45b increased apoptotic cleaved‐caspase3 fluorescence levels in the MK801+EPO+GADD45b group compared to the MK801+EPO group (Figure 6E).

The above results suggest that overexpression of GADD45b upregulated p38 MAPK activity and apoptosis in SZ. To corroborate these findings, primary hippocampal neurons were transduced with LV/GADD45b virus and treated with 10 µm of p38 MAPK inhibitor SB203580 on the ninth day. Western blotting results showed that the apoptotic cleaved‐caspase3 and phosphorylated p38 MAPK (Figure 6F–H) were reduced in the GADD45b+p38 MAPKi group compared to the GADD45b group, while SB203580 didn't affect the GADD45b levels (Figure 6F,I). mCherry was used to examine dendritic morphology (Figure 6J). Interestingly, the p38 MAPK inhibitor resulted in an obvious increase in the dendritic complexity (Figure 6K) and the total dendritic length (Figure 6L).

To further confirm these findings in SZ, primary hippocampal neurons were treated with MK801 and SB203580 on the ninth day, and the dendritic morphology of primary hippocampal neurons was examined by using anti‐MAP2 antibody (Figure S9A, Supporting Information). The results indicate that inhibition of p38 MAPK resulted in an obvious increase in the dendritic complexity and length compared to MK801 treated neurons (Figure S9B,C, Supporting Information). Western blotting showed that inhibition of p38 MAPK reduced apoptotic cleaved‐caspase3 and phosphorylated p38 MAPK to abrogate the MK801‐induced neuronal damage (Figure S9D–F, Supporting Information). In addition, p38 MAPK inhibitor didn't affect the elevation of the GADD45b levels caused by MK801 (Figure S9D,G, Supporting Information). Collectively, these data strongly support that EPO deficiency upregulated GADD45b and mediated p38 MAPK/cleaved‐caspase3 signaling pathway, resulting in synaptic and cognitive impairments in SZ.

## 3 Discussion

We have here elucidated the abnormal downregulation of EPO protein in the MK801 model of SZ, which correlates with neuronal damage in the hippocampus of SZ rats. Our findings revealed EPO deficiency in SZ rats leads to an upregulation of GADD45b. Increased GADD45b leads to elevated p38 MAPK activity and the upregulation of apoptotic proteins. Supplementation of EPO demonstrated significant improvements in synaptic damage and cognitive impairments in SZ. Thus, our study not only uncovers a novel role of EPO and GADD45b in the pathological and behavioral abnormalities associated with SZ but also identifies their potential application as therapeutic targets in SZ‐related cognitive deficits.

EPO is a cytokine that binds to the EPOR and plays a crucial role in regulating erythroid cell formation during erythropoiesis in the bone marrow.^[^ ^42^, ^43^ ^]^ However, EPO and its receptor are expressed in many other organs and tissues, including the brain, where they are primarily implicated in the regulation of tissue protection. In the central nervous system (CNS), EPO is predominantly expressed by astrocytes, while EPORs are mainly expressed in neurons. Specifically, in the CNS, EPO has been shown to promote neuroprotection against various insults such as hypoxic‐ischemic injury, ethanol‐induced neurodegeneration, epilepsy, Alzheimer's disease, and Parkinson's disease.^[^ ^44^ ^]^ In our study, EPO was significantly decreased in SZ rats caused by MK801, regarded as a surrogate for the positive symptoms of schizophrenia, and supplementation of EPO inhibited MK801‐induced synaptic and cognitive impairments, indicating EPO reduction is implicated in the pathogenesis of schizophrenia, where it leads to the upregulation of GADD45b resulted from RNA‐sequencing analysis and Western blotting in SZ rats. These findings strongly suggest a critical role of EPO implication in the pathological and behavioral changes observed in SZ.

As a vital mediator of cell death in response to cell cycle arrest, apoptosis, cell survival, and genomic stability, GADD45b is implicated in the pathogenesis of multiple neurological diseases.^[^ ^45^, ^46^, ^47^ ^]^ In line with this, we here found that GADD45b knockdown reversed MK801‐induced cognitive impairments whereas overexpression of GADD45b in wild‐type rats resulted in severe neuronal loss and synaptic dysfunction in the hippocampal CA1 region and caused cognitive deficits. Moreover, overexpression of GADD45b significantly diminished the protective effects of EPO in MK801‐induced SZ, strongly supporting that the increase in GADD45b is required for EPO deficiency‐related synaptic and cognitive deficit in SZ. Based on the KEGG analysis of RNA sequencing showing significant upregulation of p38 MAPK apoptosis pathway in MK801 rats, we further studied the p38 MAPK‐related apoptosis in SZ. Supplementation of EPO attenuates p38 MAPK apoptosis pathway in MK801 rats. However, overexpression of GADD45b blocked the anti‐apoptotic effects of EPO in MK801 rats and caused apoptosis and synaptic damage. Interestingly, our data also revealed that inhibiting p38 MAPK activity in hippocampal neurons led to the downregulation of cleaved‐caspase3 levels, thereby improving synaptic dysfunction induced by GADD45b, suggesting the implication of GADD45b/p38 MAPK in neuronal apoptosis and synaptic dysfunction.

**Figure 7:**
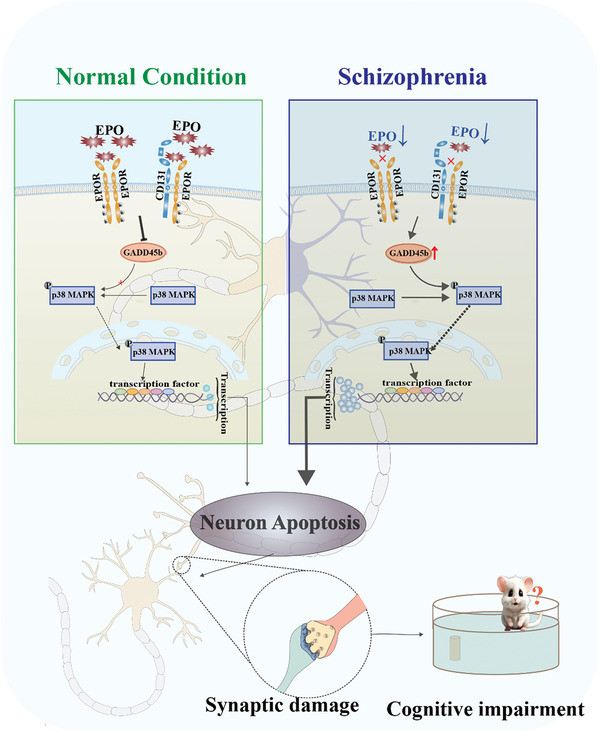
Schematic illustration of the effects of EPO in the Schizophrenia brain. EPO deficiency upregulates GADD45b triggering p38 MAPK activation and neuronal apoptosis in SZ, thus leading to synaptic damage and cognitive impairments.

In summary, the present study suggests a novel pathogenic link between SZ and cognitive impairments, where EPO deficiency upregulates GADD45b triggering p38 MAPK activation and apoptosis in SZ, thus leading to the synaptic damage and cognitive impairments (**Figure** **7**). Given the deteriorative effect of GADD45b/p38 MAPK exerts numerous apoptotic events linked to SZ and cognitive impairments, downregulation of GADD45b/p38 MAPK axis may provide a potential strategy for the treatment of SZ‐associated cognitive dysfunction.

## 4 Experimental Section

### Reagents

MK801 was obtained from Sigma Company (Product NO.M107). The p38 MAPK inhibitor SB 203580 was procured from MCE Company. Antibodies used in this study are listed in Table S1 (Supporting Information).

### Animals

Male Sprague‐Dawley rats (250 ± 20 g, 2 months old) were obtained from the Laboratory Animal Center at Tongji Medical College, Huazhong University of Science and Technology. The rats were housed under standard laboratory conditions with a 12‐h light‐dark cycle. Rats were randomly assigned to different experimental groups based on the requirements of the study. All animal procedures were conducted following the guidelines of the Institutional Animal Care and Use Committee (IACUC No. 2316) of Tongji Medical College, Huazhong University of Science and Technology, and ethical considerations were strictly adhered to.

In our study, the rats in the model (Mod) group were intraperitoneally and daily injected with MK801 (0.3 mg kg^−1^) for 1 week, while those in the control group were injected with normal saline for 1 week. The preventive (Pre) group rats were injected with AAV9/EPO virus in the hippocampal CA1 region and received daily intraperitoneal injections of MK801 (0.3 mg kg^−1^) for 1 week (Figure 1A).

To investigate whether GADD45b plays an important role in SZ‐related cognitive impairments, the rats in the control group received vector virus injections into the hippocampal CA1 region and were allowed 6 weeks for expression. Daily intraperitoneal injection with MK801 (0.3 mg kg^−1^) for 1 week was performed to establish the Mod group. The third group of rats received simultaneous Sh‐GADD45b virus injection into the hippocampal CA1 region and daily intraperitoneal injections of MK801 (0.3 mg kg^−1^) for last 1 week, serving as the MK801+Sh‐GADD45b group (Figure 4A).

To further confirm that EPO could improve SZ‐related cognitive dysfunction, which may be mediated by inhibiting the expression of GADD45b, 40 healthy SD rats were randomly divided into four groups of ten rats and treated as shown in Figure 5A. In the control group, the rats were injected with vector virus in the hippocampal CA1 region. For the Mod group, rats were daily injected intraperitoneally with MK801 (0.3 mg kg^−1^) for 1 week. The Pre group rats were simultaneously injected with AAV9/EPO virus in the hippocampal CA1 and intraperitoneally with daily MK801 (0.3 mg kg^−1^) for 1 week. In the fourth group, the rats were injected with LV/GADD45b and AAV/EPO virus in the hippocampal CA1 region and intraperitoneally with daily MK801 (0.3 mg kg^−1^) for last 1 week (MK801+EPO+GADD45b group).

### Stereotactic Surgery

The rats were anesthetized with isoflurane and positioned in the stereotactic apparatus. Following disinfection with iodophors and 75% (vol/vol) alcohol, the scalp was incised along the midline between the ears. Stereoscopic holes were drilled at stereotaxic coordinates 3.96 mm posterior, 3.0 mm lateral, and 3.0 mm ventral relative to bregma. A microinjection system from World Precision Instruments was employed to deliver AAV‐eGFP‐CMV‐EPO (2 µL, 2.0 × 10^12 viral genome per mL), Lenti‐mCherry‐CMV‐GADD45b, Lenti‐mCherry‐CMV‐shRNA (GADD45b), or Vector (2 µL, 2.0 × 10^8 viral genome per mL) into the CA1 region of the hippocampus at a rate of 0.125 µL min^−1^. Following a 10‐min infusion period, the needle was slowly withdrawn, and the incision was sutured.

### Behavior Tests—Open Field

The open‐field test was used to evaluate the autonomous exploration ability of experimental rats. The test equipment was a typical open field (100 × 100 cm^2^ PVC square arena, 70 cm high walls). The rats were allowed alone to explore the arena for 5 min and the total distance covered, and the central area crossing were tracked and measured.

### Behavior Tests—Novel Objective Recognition Test

The Novel Object Recognition test was utilized to assess rats' short‐term learning and memory abilities. Rats were introduced to the new object recognition chamber (room) 24 h before the test. Subsequently, the rats were placed in a plastic container measuring 100 × 100 × 100 cm for 5 min to familiarize themselves with the environment. Then the animals were returned to the arena from the same starting point and were given 5 min to familiarize themselves with objects A and B. Following each rat's familiarization period, the arena and objects were cleaned with 75% ethanol. Two hours after the conclusion of the familiarization period, the rats were given 5 min of exploration time, during which object B was replaced with object C. After 24 h, object C was substituted with object D, and the rats were allowed another 5 min to explore the two objects.

### Behavior Tests—Fear Conditioning

In this test, rats were placed into a square chamber measuring 40 × 40 × 50 cm. Freezing time was assessed using the Contextual NIR Video Fear Conditioning System (Med Associates). The test included two phases. The first one involved training: rats were placed in the chamber and after 3 min, a 10‐s sound stimulus was given, immediately followed by short‐term current stimulation (0.8 mA, 3 s). The current stimulation cycle was repeated three times. The second phase was the test: this phase took place either 2 or 24 h following the training and involved only sound stimulation, with no current stimulation. Freezing time was then recorded.

### Behavior Tests—Morris Water Maze (mw) Test

The Morris Water Maze was utilized to assess spatial learning and memory ability. Rats underwent training sessions four times daily over 5 days for spatial learning in a water maze aimed at locating a hidden platform. During each trial, the rats commenced from one of four quadrants facing the wall of the pool, and the trial was concluded when the rats successfully climbed onto the platform. If the rats failed to locate the platform within 60 s, they were guided to the platform and allowed to remain there for 20 s. The tracking camera recorded the time taken by the rats to swim and locate the platform (latency). Spatial memory was evaluated on the sixth or ninth day. The platform was removed, and parameters such as the number of crossings, and time spent in the target quadrant were recorded.

### Western Blotting

Primary hippocampal neurons, brain hippocampus, or hippocampus CA1 region were rapidly removed and homogenized at 4 °C in a buffer containing (in mm) 50 Tris‐HCl, pH 7.4, 150 NaCl, 10 NaF, 1 Na_3_VO_4_, 5 EDTA, 1 PMSF, and 2 benzamidine. The tissue homogenate or cell lysate was centrifuged at 12 000 rpm per min for 10 min and the supernatant was collected. The protein concentration was quantified by a bicinchoninic acid (BCA) protein kit (Pierce, Rockford, IL, USA). The proteins were separated by SDS‐polyacrylamide gel electrophoresis (10% gel) and transferred to a nitrocellulose membrane. After soaking in 5% skim milk at 25 °C for 0.5 h, the membranes were incubated with primary antibody at 4 °C overnight. The imprints were then coupled to IRDye TM (800 CW) anti‐mouse /anti‐rabbit IgG or HRP‐conjugated Goat anti‐mouse /anti‐rabbit IgG at 25 °C for 1 h, and the images were viewed with the Odyssey infrared imaging system (LI‐COR Biosciences, USA) or the ChemiScope (Clinx Science instruments Co. Ltd.).

### Primary Hippocampal Neuron Culture

Primary hippocampal neurons were prepared from Sprague‐Dawley rat embryos at 17–18 days of age. The hippocampus was dissected and lightly chopped in Hank's buffered saline solution, then suspended in 0.25% (vol/vol) trypsin solution for 12 min at 37 °C. The neurons were plated in 6‐ and 12‐well plates, which were coated with 100 µg mL^−1^ poly‐D‐lysine and supplemented with 2% (vol/vol) B‐27 and 1 × GlutaMAX, respectively. After 9 days of culture, the neurons were treated with MK801, AAV9/CTR, LV/GADD45b, and LV/Sh‐GADD45b as required by the experiment. At the end of the treatment, the cells were collected and lysed in RIPA buffer for further bioassay or fixed with 4% paraformaldehyde for immunofluorescence imaging. All cell culture reagents were purchased from Thermo Fisher Technologies. Sholl analysis is used to measure dendrite complexity. Measurement and analysis of dendritic length were performed using a semi‐automatic protocol via Imaris software (Bitplane, Inc.).

### Recording of long‐term potentiation (LTP)

The rats’ brains were quickly removed and perfused with 30 mL ice‐cold solution of aCSF containing (in mm) 120 NaCl, 2.5 KCl, 2 CaCl_2_·2H_2_O, 1.25 KH_2_PO_4_, 2 MgSO_4_·7H_2_O, 26 NaHCO_3_, and ten glucose, saturated with 95% O_2_ and 5% CO_2_ and buffered to a pH of 7.4. Following sectioning at 300–320 µm thickness, the slices were incubated in oxygenated aCSF at 32 °C and allowed to recover for 30 min and at 20–25 °C to recover for 1 h. Then slices were kept with continuous oxygenated aCSF perfusion. For LTP measurement, the slices were placed in a chamber with an 8 × 8 microelectrode array (Parker Technology, Beijing, China) on the bottom plane (each 50 × 50 mm) and soaked in aCSF. The stimulus signal was provided by the MED64 system (Alpha MED Sciences, Panasonic). The field excitatory postsynaptic potentials of CA1 neurons were recorded by stimulating CA3 neurons after three series of high‐frequency stimulation (HFS; 100 Hz, duration 1 s). The magnitude of the LTP was quantified as the percentage change in the fEPSP slope during the 60–90 min interval following LTP induction.

### mEPSCs Recording

Primary hippocampal neurons incubated in standard aCSF containing (in mm) NaCl 119, KCl 2.5, NaH_2_PO_4_ 1.25, NaHCO_3_ 26, CaCl_2_ 2.5, MgCl_2_ 1.3, D‐glucose ten saturated with 95% O_2_ and 5% CO_2_. Primary hippocampal neurons were visualized by an upright microscope (Olympus, BX51WI) and were perfused with oxygen‐saturated aCSF containing 50 µm picrotoxin and 1 µm tetrodotoxin recording at room temperature. Briefly, the micropipettes (3–5 MΩ) were tip‐filled with internal solution composed of (in mm) K‐gluconate 128, KCl 17.5, Na_2_ATP 5, MgCl_2_ 1, EGTA 0.2, and HEPES 10 (pH 7.4) and back‐filled with the same internal solution to perform the whole‐cell patch recordings for recording the mEPSC. Primary hippocampal neurons were held at −70 mV to record the mEPSC in current clamp mode. Data were accepted for analysis only if the series resistance was below 20 MΩ. Data were collected using a Multiclamp 700B patch clamp amplifier (Molecular Devices), sampled at 10 kHz, and filtered at 2 kHz using a Digidata 1440A low‐noise data acquisition system (Molecular Devices). All drugs were purchased from Sigma.

### Golgi Staining

Golgi solution preparation: Solution A: 5% potassium dichromate (dissolve 10 g potassium dichromate in 200 mL ddH₂O); Solution B: 5% mercury chloride (dissolve 10 g in 200 mL ddH₂O); Solution C: 5% potassium chromate (dissolve 8 g potassium chromate in 160 mL ddH₂O). Mix 5% potassium dichromate and 5% mercury chloride to form solution AB; mix 400 mL ddH₂O with solution C; finally, add solution AB to solution C. The final volume ratio of potassium dichromate: mercury chloride: potassium chromate: water was 5:5:4:10. Store the solution in the dark for about a week. Rats were anesthetized with isoflurane and perfused with ≈300 mL of normal saline. The brains were then removed and immersed in the Golgi staining solution for 30 days, with the Golgi solution changed every 2 days. The brains were serially sectioned into 80–100 µm‐thick slices using a vibrating microtome (Leica, VT1000S, Germany). Slices were alkalized with ammonia, dehydrated, and soaked in CXA solution for 15 min (1:1:1 mixture of 1000 mL chloroform, 1000 mL xylene, and 1000 mL anhydrous ethanol), then sealed with neutral gum and allowed to dry. The images were visualized using the high‐resolution Pathology SCAN System (Pannoramic SCAN, 3D Histech). Sholl analysis (using the cell body as the center of concentric circles and counting the intersection points with circles of different radii to reflect the number of dendritic branches). Analysis of dendritic spine: in ImageJ software, the unit dendrite length was marked, and the software automatically recorded the number of dendritic spines.

### Nissl Staining

Toluidine blue (Wuhan Haode Biotechnology Co., LTD, China) was used to stain the frozen sections, and then dehydrated with alcohol gradient (75%, 85%, 95%). The slides were cleaned twice with xylene for 5 min. The images were visualized using the high‐resolution Pathology SCAN System (Pannoramic SCAN, 3D Histech). Cell numbers were analyzed using Image software.

### TUNEL Assay

Cellular DNA fragmentation in primary hippocampal neurons was determined using TUNEL assay. Neurons were fixed in 4% paraformaldehyde for 30 min, then permeabilized in 0.5% Triton X‐100 in phosphate‐buffered saline(PBS) for 15 min. TUNEL staining was performed using one‐step TUNEL Apoptosis Assay Kit (Dalian Meilun Biotechnology Co., Ltd., Product ID: MA0224) according to the manufacturer's instructions. Fluorescence images were captured using a Zeiss LSM 710 laser scanning confocal fluorescence microscope (Zeiss, Jena, Germany) equipped with Zen software (Zeiss).

### RNA Sequencing

For the experiment, treated primary hippocampal neurons were collected in an EP tube and rapidly placed in liquid nitrogen. The samples were pre‐treated and sent to the sequencing company (MetWare Metabolic Biotechnology Co. Ltd). The transcriptome sequencing process includes RNA extraction, RNA detection, library construction, and sequencing. Experimental procedure: The samples were tested, and database construction proceeded only after passing quality checks. mRNA acquisition: mRNA with a polyA tail was enriched using Oligo(dT) magnetic beads. RNA was then fragmented using a fragmentation buffer, and first‐strand cDNA was synthesized using random six‐base hexamers from the fragmented RNA template. A buffer solution, dNTPs (dUTP, dATP, dGTP, and dCTP), and DNA polymerase I was added to synthesize double‐stranded cDNA, which was purified using AMPure XP beads. The purified double‐stranded cDNA underwent further processing. The ends were repaired, an A‐tail was added, and sequencing adapters were ligated. After library construction, the quality of the library was tested, and sequencing was conducted only if the results met the required standards. Finally, bioinformatics analysis was conducted, including differential gene expression analysis, GO analysis, and KEGG analysis.

### Transmission Electron Microscope (TEM)

Rats were anesthetized with isoflurane. After perfusion of the brain with a fixative, the hippocampus was dissected and sliced at ≈150 µm thick. The slices were further fixed by soaking in 0.1 m Na‐cacodylate buffer containing 2.5% glutaraldehyde at room temperature for 1 h. The slices were postfixed with 1% OsO_4_ to 0.1 m PBS and placed at room temperature for 2 h, then dehydrated and infiltrated. The slices were photographed under a light microscope and then continuously cut into semithin (2 µm thick) slices, then stained with 1% toluidine blue in 1% sodium borate, and the location of the CA1 region was examined under a light microscope. The selected semithin slices were further cut into a series of ultra‐thin slices by using the Leica ultramicrotome. The ultrathin sections were examined by Hitachi HT7800 transmission electron microscopy. The synaptic density, presynaptic vesicles, and the length of postsynaptic densities were measured.

### Real‐Time Quantitative PCR (QPCR)

Brain tissues were isolated using Trizol reagent following the manufacturer's instructions (Invitrogen, Carlsbad, CA, USA). A reverse transcription kit (Takara, Dalian, China) was used to reverse transcribed into cDNA. The primers for GADD45b sense: CCTCCTGGTCACGAACTGTC antisense: GGACCCACTGGTTATTGCCT, GADD45 g sense: TTGCATCCTCATTTCGAACCC antisense: GGCTCTCCTCGCAGAACAA, and GAPDH sense: TGCCTTCTCTTGTGACAAAGTGG antisense: CATTGCTGACAATCTTGAGGGAG were listed. The PCR cycle included an initial denaturation step at 95 °C for 30 s, followed by 40 cycles of denaturation at 95 °C for 5 s, annealing at 60 °C for 30 s, and extension at 72 °C for 30 s. Melting curve analysis was conducted after each experiment. Amplification and analysis were carried out using a Step One Plus real‐time PCR detection system (Life Technologies, NY, USA).

### Fluorescence Imaging and Confocal Microscopy

Brain slices or primary hippocampal neurons were fixed in 4% paraformaldehyde and permeabilized at room temperature in PBS containing 0.5% TritonX‐100 for 30 min. Following permeabilization, brain slices or primary hippocampal neurons were blocked with 5% bovine serum albumin (BSA) for 60 min. They were subsequently incubated with the primary antibody at 4 °C overnight, followed by incubation with CoraLite488, 594, 647‐conjugated secondary antibody at 37 °C for 1 h. Nuclear staining was achieved using DAPI at a concentration of 0.1 µg mL^−1^. All fluorescence images were captured using a Zeiss LSM 710 laser scanning confocal fluorescence microscope (Zeiss, Jena, Germany) equipped with Zen software (Zeiss). Image‐Pro Plus software (Media Cybernetics, CA, USA) was utilized for image analysis.

### Statistical Analysis

Data were presented as the mean ± SD and analyzed using GraphPad Prism 8.0 statistical software (https://www.graphpad.com/scientific‐software/prism/). A two‐tailed *t*‐test was used to assess the variance between two groups, and the difference among multiple groups was determined using one‐ or two‐way analysis of variance (ANOVA) with Tukey's multiple or Sidak's multiple comparisons test. *p‐*value < 0.05 was considered statistically significant. Statistical details are provided in Table S2 (Supporting Information).

## Conflict of Interest

The authors declare no conflict of interest.

## Ethics Approval and Consent to Participate

No humans were used in this research. All animal experiments were approved by the Animal Care and Use Committee of Huazhong University of Science and Technology and performed in compliance with the National Institutes of Health Guide for the Care and Use of Laboratory Animals.

## Author Contributions

C.G., W.L., and Y.L. contributed equally to this work. X.W. designed, planned, and organized all experiments and results, including the writing of the manuscript. S.S.L. and C.P.G. planned and performed all experiments. W.S.L., Y.L., X.Q.T., and Y.A.R.M. assisted with the manuscript preparation. R.L. and J.W. analyzed and interpreted the data. All authors read and approved the final manuscript.

## Supporting information



Supporting Information

## Data Availability

The data that support the findings of this study are available from the corresponding author upon reasonable request.

## References

[1] S. Jauhar , M. Johnstone , P. J. McKenna , Lancet 2022, 399, 473. 35093231 10.1016/S0140-6736(21)01730-X

[2] R. A. McCutcheon , T. Reis Marques , O. D. Howes , JAMA Psychiatry 2020, 77, 201. 31664453 10.1001/jamapsychiatry.2019.3360

[3] B. R. Rund , Scand. J. Psychol. 2018, 59, 49. 29356007 10.1111/sjop.12414

[4] F. C. Nucifora Jr. , E. Woznica , B. J. Lee , N. Cascella , A. Sawa , Neurobiol. Dis. 2019, 131, 104257. 30170114 10.1016/j.nbd.2018.08.016 PMC6395548

[5] C. B. Pedersen , O. Mors , A. Bertelsen , B. L. Waltoft , E. Agerbo , J. J. McGrath , P. B. Mortensen , W. W. Eaton , JAMA Psychiatry 2014, 71, 573. 24806211 10.1001/jamapsychiatry.2014.16

[6] P. Stepnicki , M. Kondej , A. A. Kaczor , Molecules 2018, 23, 2087. 30127324 10.3390/molecules23082087 PMC6222385

[7] H. El Kirat , A. Khattabi , M. Khalis , Z. Belrhiti , Schizophr. Res. 2023, 262, 112. 37948884 10.1016/j.schres.2023.10.021

[8] L. Baandrup , Basic Clin. Pharmacol. Toxicol. 2020, 126, 183. 31908124 10.1111/bcpt.13384

[9] M. J. Owen , A. Sawa , P. J. McKenna , Lancet 2016, 388, 86. 26777917 10.1016/S0140-6736(15)01121-6 PMC4940219

[10] M. Zink , C. U. Correll , Expert Rev. Clin. Pharmacol. 2015, 8, 335. 25916667 10.1586/17512433.2015.1040393

[11] O. Shahin , S. M. Gohar , W. Ibrahim , S. M. El‐Makawi , W. Fakher , D. B. Taher , M. Abdel Samie , M. A. Khalil , A. A. Saleh , Int. J .Psychiatry Clin. Pract. 2022, 26, 370. 35192426 10.1080/13651501.2022.2035770

[12] A. Ventriglio , A. Bellomo , F. Ricci , G. Magnifico , A. Rinaldi , L. Borraccino , C. Piccininni , F. Cuoco , G. Gianfelice , M. Fornaro , S. Delle Monache , D. De Berardis , Curr. Top. Med. Chem. 2021, 21, 1500. 34218785 10.2174/1568026621666210701103147

[13] I. Hassouna , C. Ott , L. Wüstefeld , N. Offen , R. A. Neher , M. Mitkovski , D. Winkler , S. Sperling , L. Fries , S. Goebbels , I. C. Vreja , N. Hagemeyer , M. Dittrich , M. F. Rossetti , K. Kröhnert , K. Hannke , S. Boretius , A. Zeug , C. Höschen , T. Dandekar , E. Dere , E. Neher , S. O. Rizzoli , K.‐A. Nave , A.‐L. Sirén , H. Ehrenreich , Mol. Psychiatry 2016, 21, 1752. 26809838 10.1038/mp.2015.212 PMC5193535

[14] X.‐B. Li , W. Zheng , Y.‐P. Ning , D.‐B. Cai , X.‐H. Yang , G. Ungvari , C. Ng , C.‐Y. Wang , Y.‐T. Xiang , Pharmacopsychiatry 2018, 51, 100. 28718181 10.1055/s-0043-114670

[15] A. Y. Galvez‐Contreras , T. Campos‐Ordonez , V. Lopez‐Virgen , J. Gomez‐Plascencia , R. Ramos‐Zuniga , O. Gonzalez‐Perez , Cytokine Growth Factor Rev. 2016, 32, 85. 27618303 10.1016/j.cytogfr.2016.08.004

[16] D. Wakhloo , F. Scharkowski , Y. Curto , U. Javed Butt , V. Bansal , A. A. Steixner‐Kumar , L. Wüstefeld , A. Rajput , S. Arinrad , M. R. Zillmann , A. Seelbach , I. Hassouna , K. Schneider , A. Q. Ibrahim , H. B. Werner , H. Martens , K. Miskowiak , S. M. Wojcik , S. Bonn , J. Nacher , K.‐A. Nave , H. Ehrenreich , Nat. Commun. 2020, 11, 1313. 32152318 10.1038/s41467-020-15041-1 PMC7062779

[17] S. Suresh , P. K. Rajvanshi , C. T. Noguchi , Front. Physiol. 2019, 10, 1534. 32038269 10.3389/fphys.2019.01534 PMC6984352

[18] B. Schuler , J. Vogel , B. Grenacher , R. A. Jacobs , M. Arras , M. Gassmann , FASEB J. 2012, 26, 3884. 22683849 10.1096/fj.11-191197

[19] D. Ostrowski , R. Heinrich , J. Clin. Med. 2018, 7, 24. 29393890 10.3390/jcm7020024 PMC5852440

[20] K. W. Miskowiak , M. Vinberg , J. Macoveanu , H. Ehrenreich , N. Køster , B. Inkster , O. B. Paulson , L. V. Kessing , A. Skimminge , H. R. Siebner , Biol. Psychiatry 2015, 78, 270. 25641635 10.1016/j.biopsych.2014.12.013

[21] C. C. Hernandez , C. F. Burgos , A. H. Gajardo , T. Silva‐Grecchi , J. Gavilan , J. R. Toledo , J. Fuentealba , Neural Regen. Res. 2017, 12, 1381. 29089974 10.4103/1673-5374.215240 PMC5649449

[22] F. Rey , A. Balsari , T. Giallongo , S. Ottolenghi , A. M. Di Giulio , M. Samaja , S. Carelli , ASN Neuro 2019, 11, 175909141987142. 10.1177/1759091419871420 PMC6712762 31450955

[23] M. Brines , A. Cerami , Nat. Rev. Neurosci. 2005, 6, 484. 15928718 10.1038/nrn1687

[24] A. L. Siren , T. Fasshauer , C. Bartels , H. Ehrenreich , Neurotherapeutics 2009, 6, 108. 19110203 10.1016/j.nurt.2008.10.041 PMC5084260

[25] H. Ehrenreich , M. Hasselblatt , F. Knerlich , N. von Ahsen , S. Jacob , S. Sperling , H. Woldt , K. Vehmeyer , K.‐A. Nave , A.‐L. Sirén , Proc. Natl. Acad. Sci. USA 2005, 102, 862. 15642952 10.1073/pnas.0406008102 PMC545528

[26] M. Digicaylioglu , S. Bichet , H. H. Marti , R. H. Wenger , L. A. Rivas , C. Bauer , M. Gassmann , Proc. Natl. Acad. Sci. USA 1995, 92, 3717. 7731971 10.1073/pnas.92.9.3717 PMC42032

[27] H. H. Marti , R. H. Wenger , L. A. Rivas , U. Straumann , M. Oigicaylioglu , V. Henn , Y. Yonekawa , C. Bauer , M. Gassmann , Eur. J. Neurosci. 1996, 8, 666. 9081618 10.1111/j.1460-9568.1996.tb01252.x

[28] M. Hereta , K. Kaminska , Z. Rogoz , Pharmacol. Rep. 2019, 71, 768. 31351318 10.1016/j.pharep.2019.04.007

[29] A. K. Kraeuter , T. Mashavave , A. Suvarna , M. van den Buuse , Z. Sarnyai , Psychopharmacology (Berl) 2020, 237, 1397. 31993694 10.1007/s00213-020-05467-2

[30] C. Guo , Y. Liu , M.‐S. Fang , Y. Li , W. Li , Y. A. R. Mahaman , K. Zeng , Y. Xia , D. Ke , R. Liu , J.‐Z. Wang , H. Shen , X. Shu , X. Wang , Neurotherapeutics 2020, 17, 1271. 32367475 10.1007/s13311-020-00859-w PMC7609637

[31] H. Wu , X. Wang , Y. Gao , F. Lin , T. Song , Y. Zou , L. Xu , H. Lei , Neuroscience 2016, 322, 221. 26917273 10.1016/j.neuroscience.2016.02.043

[32] E. F. Wagner , A. R. Nebreda , Nat. Rev. Cancer 2009, 9, 537. 19629069 10.1038/nrc2694

[33] D. R. Green , F. Llambi , Cold Spring Harb. Perspect. Biol. 2015, 7, a006080. 26626938 10.1101/cshperspect.a006080 PMC4665079

[34] J. Yue , J. M. Lopez , Int. J. Mol. Sci. 2020, 21, 2346.32231094

[35] K. Patel , M. G. Murray , K. A. Whelan , Adv. Exp. Med. Biol. 2022, 1360, 23. 35505160 10.1007/978-3-030-94804-7_2

[36] N. Azam , M. Vairapandi , W. Zhang , B. Hoffman , D. A. Liebermann , J. Biol. Chem. 2001, 276, 2766. 11022036 10.1074/jbc.M005626200

[37] J. M. Kearsey , P. J. Coates , A. R. Prescott , E. Warbrick , P. A. Hall , Oncogene 1995, 11, 1675. 7478594

[38] W. Fan , G. Richter , A. Cereseto , C. Beadling , K. A. Smith , Oncogene 1999, 18, 6573. 10597261 10.1038/sj.onc.1203054

[39] M. Takekawa , H. Saito , Cell 1998, 95, 521. 9827804 10.1016/s0092-8674(00)81619-0

[40] Y. R. Chen , C. F. Meyer , T. H. Tan , J. Biol. Chem. 1996, 271, 631. 8557665 10.1074/jbc.271.2.631

[41] D. V. Bulavin , O. Kovalsky , M. C. Hollander , A. J. Fornace Jr. , Mol. Cell. Biol. 2003, 23, 3859. 12748288 10.1128/MCB.23.11.3859-3871.2003 PMC155214

[42] A. S. Tsiftsoglou , Cells 2021, 10, 2140.34440909

[43] D. Gilboa , Y. Haim‐Ohana , N. Deshet‐Unger , N. Ben‐Califa , S. Hiram‐Bab , D. Reuveni , E. Zigmond , M. Gassmann , Y. Gabet , C. Varol , D. Neumann , Sci. Rep. 2017, 7, 10379. 28871174 10.1038/s41598-017-11082-7 PMC5583293

[44] D. Sargin , H. Friedrichs , A. El‐Kordi , H. Ehrenreich , Best Pract. Res. Clin. Anaesthesiol. 2010, 24, 573. 21619868 10.1016/j.bpa.2010.10.005

[45] H.‐Y. Park , Y.‐K. Ryu , Y.‐H. Kim , T.‐S. Park , J. Go , J. H. Hwang , D.‐H. Choi , M. Rhee , C.‐H. Lee , K.‐S. Kim , Neurobiol. Dis. 2016, 89, 169. 26875664 10.1016/j.nbd.2016.02.013

[46] K. Zhang , Q. Zhang , J. Deng , J. Li , J. Li , L. Wen , J. Ma , C. Li , Cell Death Dis. 2019, 10, 360. 31043581 10.1038/s41419-019-1596-z PMC6494915

[47] D. Grassi , H. Franz , R. Vezzali , P. Bovio , S. Heidrich , F. Dehghanian , N. Lagunas , C. Belzung , K. Krieglstein , T. Vogel , Cereb Cortex 2017, 27, 4166. 28444170 10.1093/cercor/bhx095

