# Supplementary material for: EPO Deficiency Upregulates GADD45b/p38 MAPK Axis, Mediating Schizophrenia‐Related Synaptic and Cognitive Impairments
Source: Adv Sci (Weinh). 2024 Oct 28;11(47):2406979. doi: 10.1002/advs.202406979 (PMC11653605; doi:10.1002/advs.202406979)
Supplement: Supplementary file 1 — Supporting Information [file ADVS-11-2406979-s001.docx]

**Supplementary Figure 1**

**
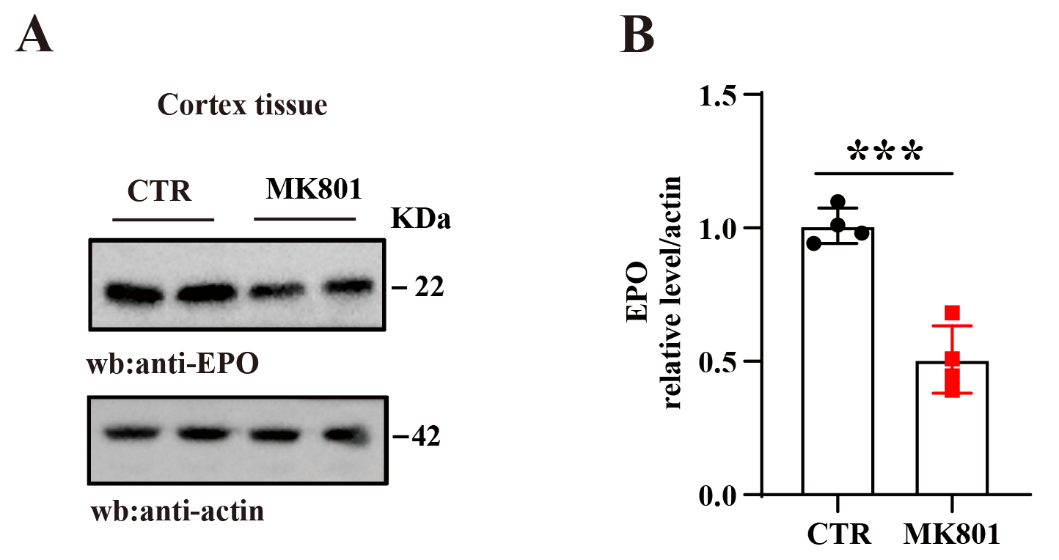
**

**Figure S1. EPO levels of the cortex were decreased in MK801 rats.**

(A) Brain tissues (cortex) from the control and MK801 groups were homogenized. EPO protein levels were detected by immunoblotting, actin was used as a loading control. (B) Quantitative analysis of the EPO, (n = 4). Data are presented as Mean ± SD. ***p < 0.001, versus Control group. Statistical details were provided in Table S2.

**Supplementary Figure 2**

**
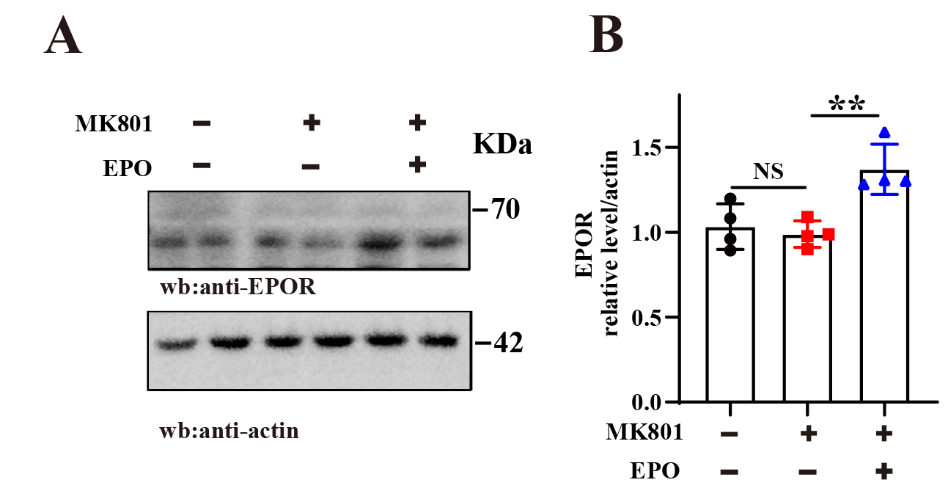
**

**Figure S2****. EPOR levels were increased by supplementation of EPO in schizophrenia model**.

(A) Brain tissues (hippocampus) from the three groups were homogenized and EPOR protein levels were detected by immunoblotting. Actin was used as a loading control. (B) Quantitative analysis of the EPOR, (n = 4). Data are presented as Mean ± SD. **p < 0.01, versus MK801 group. Statistical details were provided in Table S2.

**Supplementary Figure 3**

**
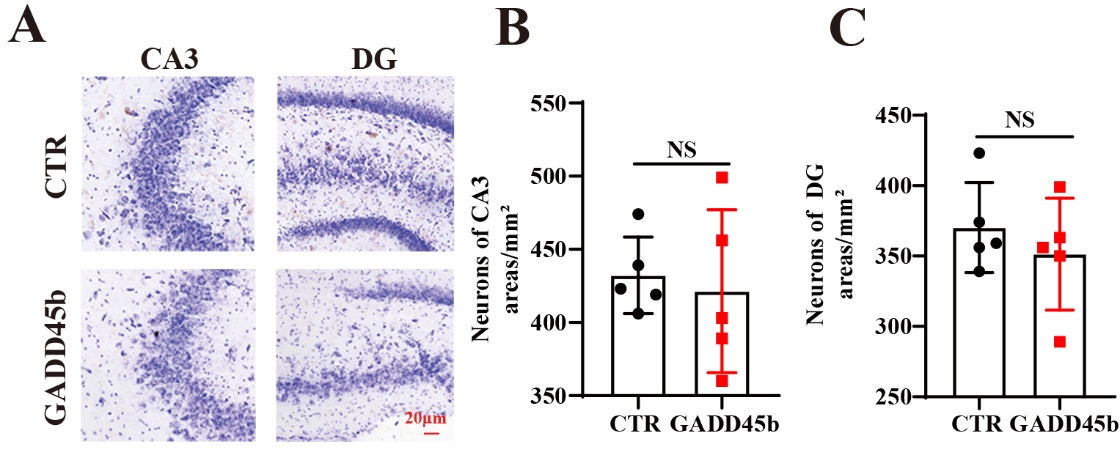
**

**Figure S3. The number of neurons in the hippocampal CA3 and DG regions were no significant difference following GADD45b overexpression.**

(A) Representative Nissl staining of the hippocampus, (scale bar: 20 μm). The quantitative analysis of the number of neurons in the hippocampal CA3 (B) and DG (C) regions were conducted, (n=5). Data are presented as Mean ± SD. Statistical details were provided in Table S2.

**Supplementary Figure 4**

**
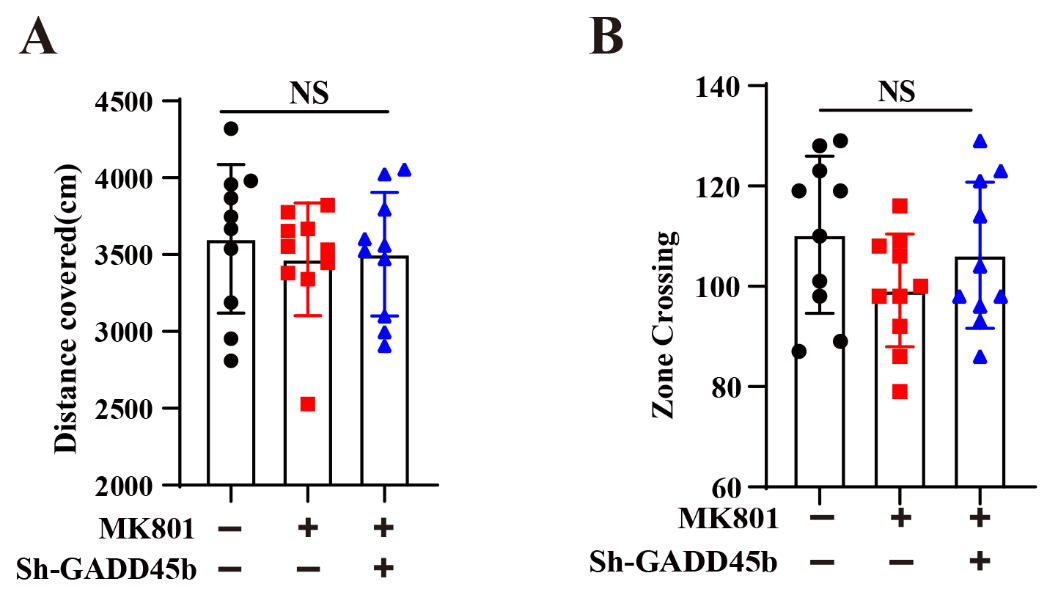
**

**Figure S4.** **Open field showed no significant difference following GADD45b overexpression.**

The open field test showed the total distance covered (A) and the zone crossing (B), (n =10). Data are presented as Mean ± SD. Statistical details were provided in Table S2.

**Supplementary Figure 5**

**
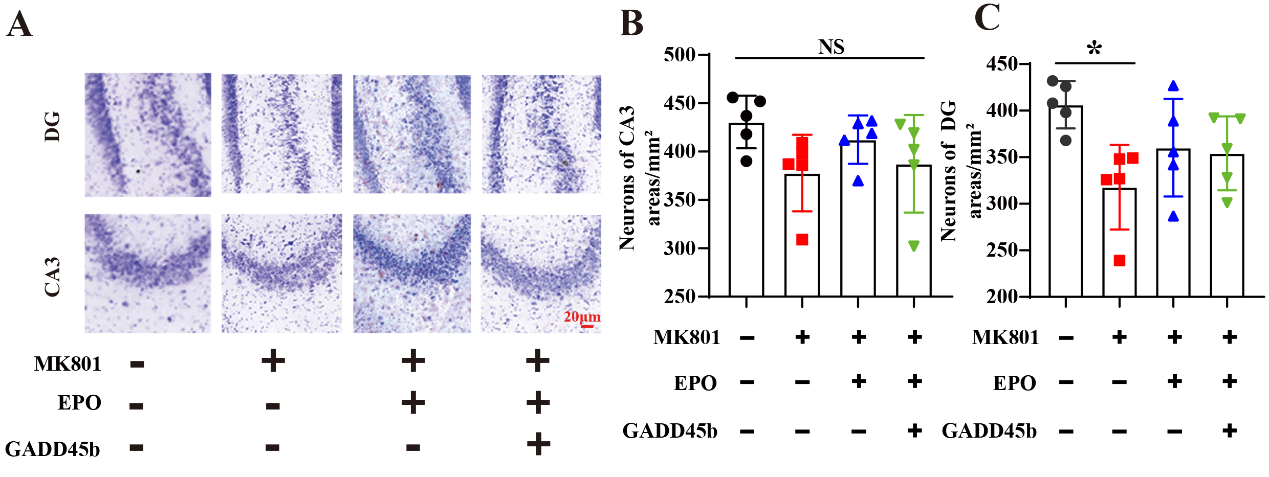
**

**Figure S5. The number of neurons in the hippocampal CA3 and DG regions were no significant difference in the MK801+EPO+GADD45b and MK801+EPO group.**

(A) Representative Nissl staining of the hippocampus, (scale bar: 20 μm). The quantitative analysis of the number of neurons in the hippocampal CA3 (B) and DG (C) regions were conducted, (n=5). Data are presented as Mean ± SD. *p < 0.05, versus Control group. Statistical details were provided in Table S2.

**Supplementary Figure 6**

**
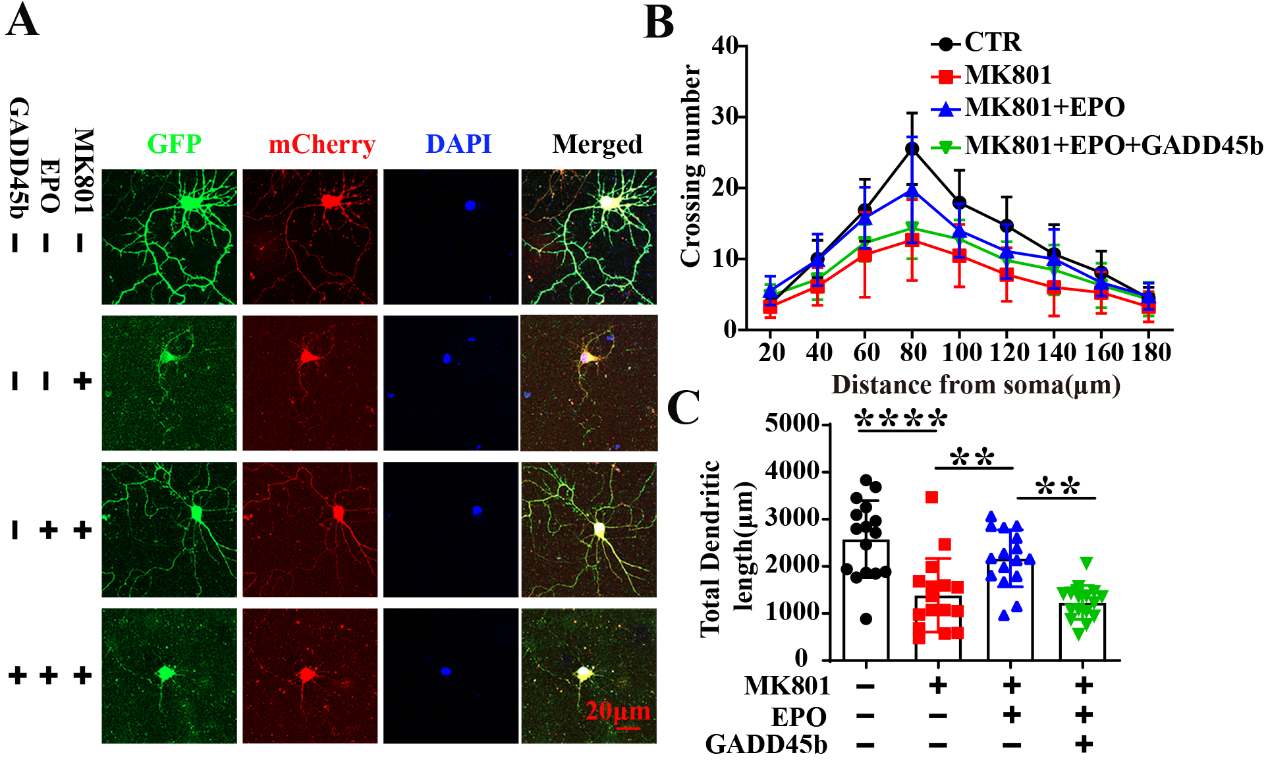
**

**Figure S6. Overexpression of GADD45b blocked the protective effects of EPO in primary hippocampal neurons.**

(A) Rats' primary hippocampal neurons were transduced with either AAV9/EPO or LV/GADD45b virus and treated with MK801 on the ninth day. GFP and mCherry were utilized to assess dendritic morphology. Representative images are shown post-treatment (A), (scale bar: 20 μm). Sholl analysis (B), quantitative analyses of dendritic length (C), (n = 16 hippocampal neurons). Data are presented as Mean ± SD. **p < 0.01, ****p < 0.0001, versus MK801or MK801+EPO group. Statistical details were provided in Table S2.

**Supplementary Figure 7**

**
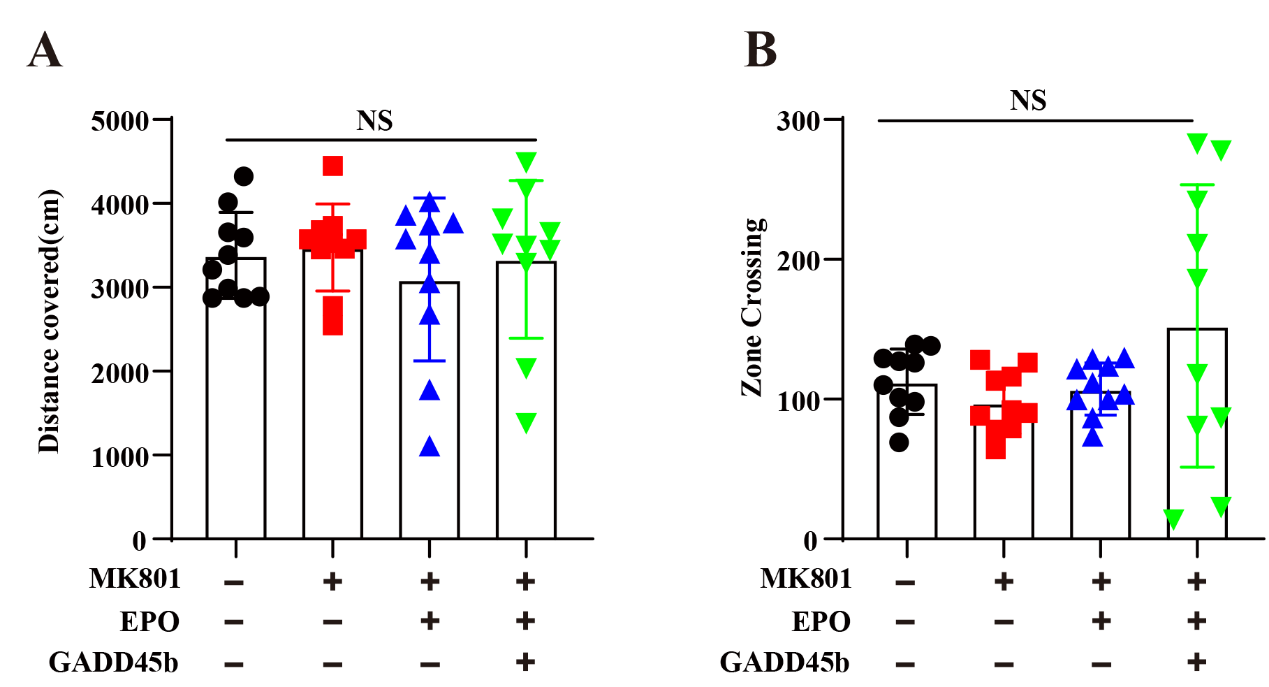
**

**Figure S7. Open field showed no significant difference in the MK801+EPO+GADD45b and MK801+EPO group.**

The open field test showed the total distance covered (A) and the zone crossing (B), (n =10). Data are presented as Mean ± SD. Statistical details were provided in Table S2.

**Supplementary Figure 8**

**
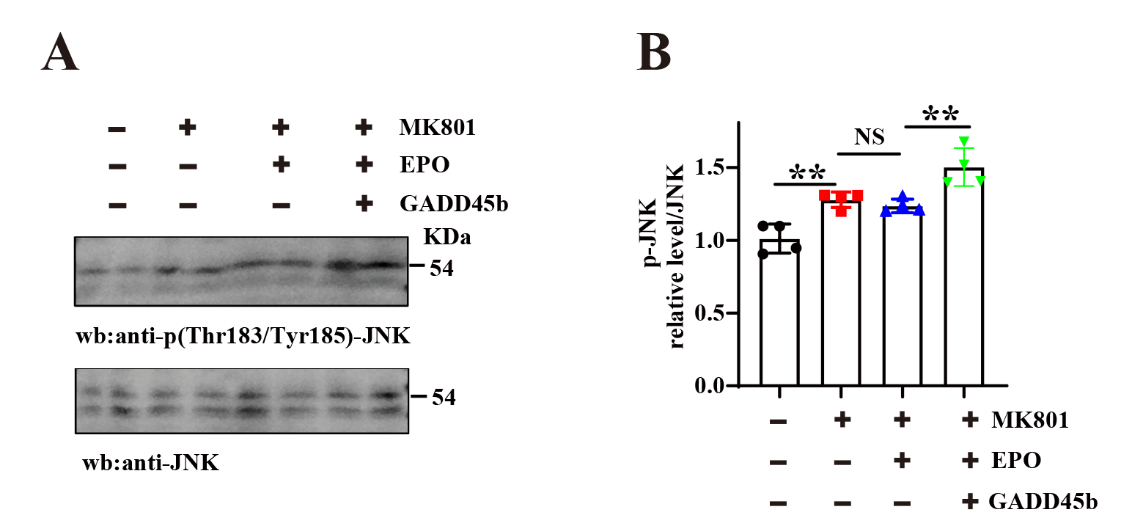
**

**Figure S8. p-JNK/JNK levels were no significant difference by supplementation with EPO in schizophrenia model.**

(A) Brain tissues (hippocampus) from the four groups were homogenized and p-JNK/JNK protein levels were detected by immunoblotting. (B) Quantitative analysis of the p-JNK/JNK, (n = 4). Data are presented as Mean ±SD. **p < 0.01, versus MK801 or MK801+EPO group. Statistical details were provided in Table S2.

**Supplementary Figure 9**

**
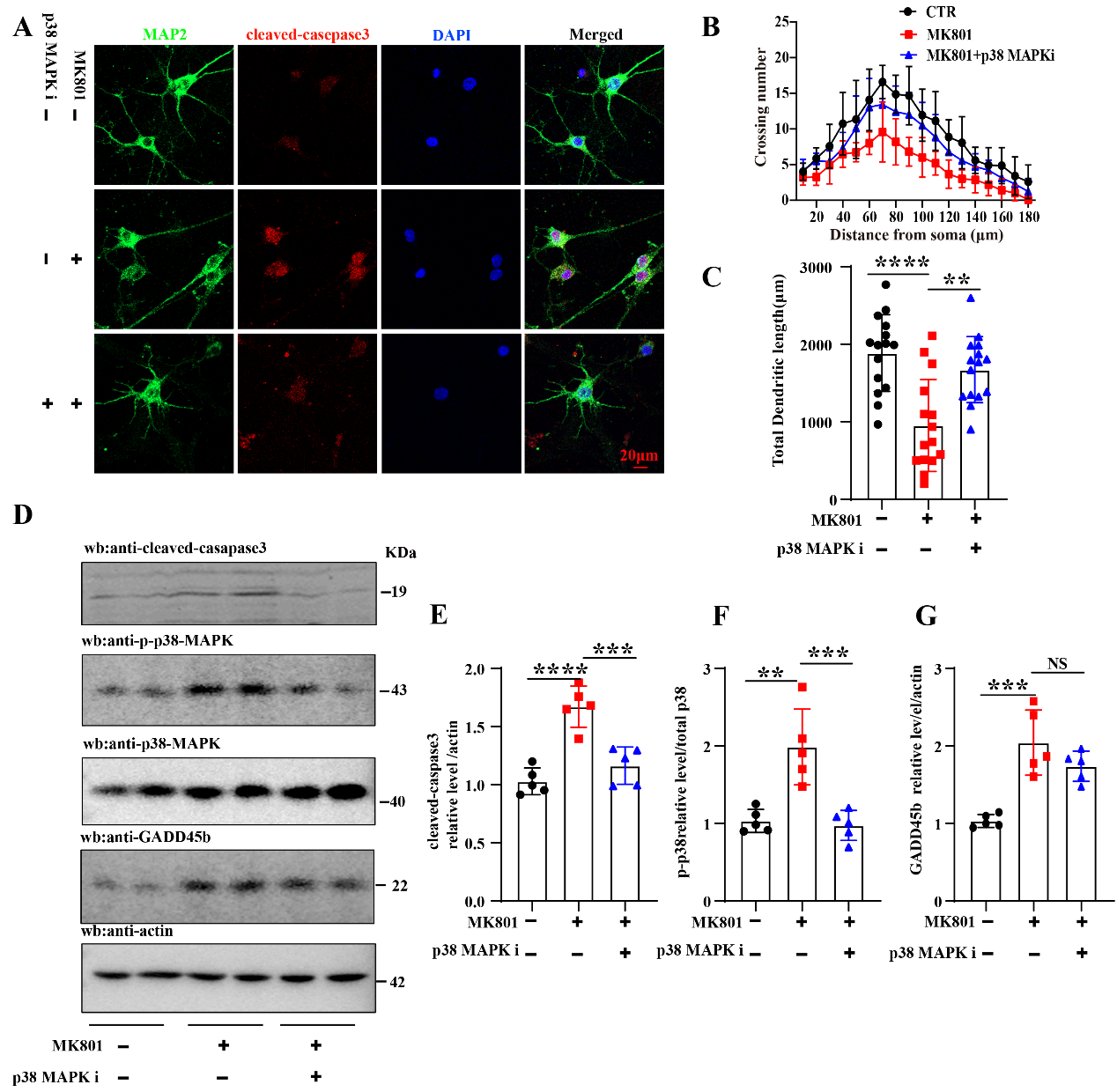
**

**Figure S9. Inhibition of p38 MAPK improved MK801-induced synaptic damage in primary hippocampal neurons.**

(A) Rats’ primary hippocampal neurons were treated with MK801 and p38 MAPK inhibitor on the ninth day. The dendritic morphology of hippocampal primary neurons was examined by using an anti-MAP2 antibody, and the cleaved-caspase3 was measured by immunofluorescence (A), (scale bar: 20 μm). Sholl analysis (B), quantitative analyses of dendritic length (C), (n = 15 hippocampal neurons). (D) Primary hippocampal neurons were homogenized, and GADD45b, p-p38 MAPK/p38 MAPK, and cleaved-caspase3 protein levels were detected by immunoblotting, actin was used as a loading control. Quantitative analysis of the cleaved-caspase3 (E), p-p38 MAPK/p38 MAPK (F), and GADD45b (G), (n=5). Data are presented as Mean ± SD. **p < 0.01, ***p < 0.001, ****p < 0.0001, versus MK801 group. Statistical details were provided in Table S2.

**Table S1. Antibodies used in this study.**

| Antibodies | | SOURCE | | IDENTIFIER | |
| --- | --- | --- | --- | --- | --- |
| Beta Actin | 1:2000 for WB | | Proteintech | | 66009-1-Ig |
| Cleaved caspase 3 | 1:1000 for WB  1:200 for IF | | Cell signaling | | #9661 |
| p38 MAPK (D13E1) | 1:1000 for WB, | | Cell signaling | | #8690 |
| Phospho-p38 MAPK (Thr180/Tyr182) (D3F9) | 1:1000 for WB | | Cell signaling | | #4511 |
| Erythropoietin | 1:1000 for WB | | Atagenix | | ATMA10247Mo |
| Erythropoietin Receptor | 1:500 for WB | | ZENBIO | | 860313 |
| GADD45b Antibody | 1:1000 for WB | | Thermofisher | | PA5-100741 |
| JNK | 1:1000 for WB | | Cell signaling | | #9252 |
| Phospho-SAPK/JNK (Thr183/Tyr185) (81E11) | 1:500 for WB | | Cell signaling | | #4668 |
| MAP2 | 1:300 for IF | | Sigma | | [M9942](https://www.sigmaaldrich.cn/CN/zh/product/sigma/m9942) |
| CoraLite594 – conjugated Goat Anti-Mouse | 1:200 for IF | | Proteintech | | SA00013-3 |
| CoraLite488-conjugated Goat Anti-Rabbit IgG(H+L) | 1:200 for IF | | Proteintech | | SA00013-2 |
| CoraLite647-conjugated AffiniPure F(ab')2 Fragment Goat Anti-Rabbit IgG (H+L) | 1:200 for IF | | Proteintech | | SA00014-9 |
| HRP-conjugated Goat anti-Mouse IgG (H+L) | 1:10,000 for WB | | Abclonal | | AS003 |
| HRP-conjugated Goat anti-Rabbit IgG (H+L) | 1:10,000 for WB | | Abclonal | | AS014 |
| IRDye 800CW Goat anti-Mouse IgG | 1:10,000 for WB | | LI-COR Biosciences | | AB_621842 |
| IRDye 800CW Goat anti-Rabbit IgG | 1:10,000 for WB | | LI-COR Biosciences | | AB_621843 |

WB:Wesrern blotting; IF:Immunofluorescence

**Table S2. Statistical analysis for Figure 1-6 and Figure S1-9**

| Figure | Statistic method | Number(n) | Statistic results | Value |
| --- | --- | --- | --- | --- |
| 1D | One-way ANOVA with Tukey's multiple comparisons test | 5,5,5 | P<0.0001 | F (2, 12) = 27.45 |
| 1F | One-way ANOVA with Tukey's multiple comparisons test | 5,5,5 | P=0.0002 | F (2, 12) = 18.43 |
| 1I | One-way ANOVA with Tukey's multiple comparisons test | 20,20,20 | P<0.0001 | F (2, 57) = 21.92 |
| 1J | One-way ANOVA with Tukey's multiple comparisons test | 35,35,35 | P<0.0001 | F (2, 102) = 27.57 |
| 1L | One-way ANOVA with Tukey's multiple comparisons test | 10,10,10 | P=0.0010 | F (2, 27) = 8.979 |
| 1M | One-way ANOVA with Tukey's multiple comparisons test | 50,50,50 | P<0.0001 | F (2, 147) = 36.41 |
| 1N | One-way ANOVA with Tukey's multiple comparisons test | 50,50,50 | P=0.2247 | F (2, 147) = 1.508 |
| 1O | One-way ANOVA with Tukey's multiple comparisons test | 11,12,12 | P=0.7237 | F (2, 32) = 0.3266 |
| 1P | One-way ANOVA with Tukey's multiple comparisons test | 11,12,12 | P=0.7852 | F (2, 32) = 0.2437 |
| 1Q | One-way ANOVA with Tukey's multiple comparisons test | 11,12,12 | P=0.0023 | F (2, 32) = 7.372 |
| 1R | One-way ANOVA with Tukey's multiple comparisons test | 11,12,12 | P=0.0028 | F (2, 32) = 7.118 |
| 1S | One-way ANOVA with Tukey's multiple comparisons test | 11,12,12 | P=0.0017 | F (2, 32) = 7.856 |
| 1T | One-way ANOVA with Tukey's multiple comparisons test | 11,12,12 | P=0.0010 | F (2, 32) = 8.638 |
| 1V | Two-way ANOVA with Tukey's multiple comparisons test | 11,11,11 | P=0.1745  P<0.0001  P<0.0001 | F (8, 150) = 1.465  F (4, 150) = 43.25  F (2, 150) = 14.59 |
| 1W | One-way ANOVA with Tukey's multiple comparisons test | 11,11,11 | P=0.0010 | F (2, 30) = 8.844 |
| 1X | One-way ANOVA with Tukey's multiple comparisons test | 11,11,11 | P=0.0084 | F (2, 30) = 5.632 |
| 2C | One-way ANOVA with Tukey's multiple comparisons test | 15,15,15 | P=0.0031 | F (2, 42) = 6.629 |
| 2E | One-way ANOVA with Tukey's multiple comparisons test | 8,10,10;  8,10,10 | P=0.0118;  P=0.9124 | F (2, 25) = 5.325;  F (2, 25) = 0.09199 |
| 2I | One-way ANOVA with Tukey's multiple comparisons test | 5,5,5 | P=0.0001 | F (2, 12) = 21.72 |
| 2J | One-way ANOVA with Tukey's multiple comparisons test | 5,5,5 | P=0.0019 | F (2, 12) = 11.11 |
| 2K | One-way ANOVA with Tukey's multiple comparisons test | 5,5,5 | P=0.0010 | F (2, 12) = 12.90 |
| 2L | One-way ANOVA with Tukey's multiple comparisons test | 5,5,5 | P=0.0003 | F (2, 12) = 17.42 |
| 3D | Two-tailed t test | 5,5 | P=0.0003 | t=6.233, df=8 |
| 3F | Two-tailed t test | 5,5 | P=0.0047 | t=3.874, df=8 |
| 3I | Two-tailed t test | 15,15 | P<0.0001 | t=8.476, df=28 |
| 3J | Two-tailed t test | 50,50 | P<0.0001 | t=7.432, df=98 |
| 3L | Two-tailed t test | 10,10 | P<0.0001 | t=7.494, df=18 |
| 3M | Two-tailed t test | 50,50 | P=0.0002 | t=3.898, df=98 |
| 3N | Two-tailed t test | 50,50 | P=0.6825 | t=0.4103, df=98 |
| 3P | Two-tailed t test | 5,5 | P=0.0069 | t=3.610, df=8 |
| 3Q | Two-tailed t test  Two-tailed t test | 9,9  9,9 | P=0.8980  P= 0.0332 | t=0.1303, df=16  t=2.331, df=16 |
| 3R | Two-tailed t test  Two-tailed t test | 9,9  9,9 | P= 0.8904  P= 0.0024 | t=0.1400, df=16  t=3.608, df=16 |
| 3S | Two-way ANOVA with Sidak's multiple comparisons test | 9,9 | \| P=0.2106 \| \| --- \| \| P<0.0001 \| \| P=0.0009 \| | \| F (4, 80) = 1.498 \| \| --- \| \| F (4, 80) = 71.40 \| \| F (1, 80) = 11.87 \| |
| 3U | Two-tailed t test | 9,9 | P=0.0348  P=0.2196 | t=2.307, df=16  t=1.278, df=16 |
| 3V | Two-tailed t test | 9,9 | P=0.0271  P=0.0193 | t=2.432, df=16  t=2.600, df=16 |
| 4D | One-way ANOVA with Tukey's multiple comparisons test | 5,5,5 | P=0.0003 | F (2, 12) = 17.08 |
| 4F | One-way ANOVA with Tukey's multiple comparisons test | 5,5,5 | P=0.0001 | F (2, 12) = 21.70 |
| 4I | One-way ANOVA with Tukey's multiple comparisons test | 20,20,20 | P<0.0001 | F (2, 57) = 11.64 |
| 4J | One-way ANOVA with Tukey's multiple comparisons test | 50,50,50 | P<0.0001 | F (2, 147) = 26.46 |
| 4L | One-way ANOVA with Tukey's multiple comparisons test | 10,10,10 | P=0.0002 | F (2, 27) = 11.85 |
| 4M | One-way ANOVA with Tukey's multiple comparisons test | 50,50,50 | P=0.0018 | F (2, 147) = 6.597 |
| 4N | One-way ANOVA with Tukey's multiple comparisons test | 50,50,50 | P=0.8547 | F (2, 147) = 0.1571 |
| 4P | One-way ANOVA with Tukey's multiple comparisons test | 5,5,5 | P=0.0016 | F (2, 12) = 11.62 |
| 4Q | One-way ANOVA with Tukey's multiple comparisons test | 10,10,10 | P=0.0004 | F (2, 27) = 10.56 |
| 4R | One-way ANOVA with Tukey's multiple comparisons test | 10,10,10 | P<0.0001 | F (2, 27) = 19.21 |
| 4S | Two-way ANOVA with Tukey's multiple comparisons test | 10,10,10 | P=0.2732  P<0.0001  P<0.0001 | F (8, 135) = 1.254  F (4, 135) = 63.20  F (2, 135) = 13.72 |
| 4T | One-way ANOVA with Tukey's multiple comparisons test | 10,10,10 | P=0.0065 | F (2, 27) = 6.100 |
| 4U | One-way ANOVA with Tukey's multiple comparisons test | 10,10,10 | P=0.0011 | F (2, 27) = 8.820 |
| 5D | One-way ANOVA with Tukey's multiple comparisons test | 5,5,5,5 | P<0.0001 | F (3, 16) = 30.57 |
| 5E | One-way ANOVA with Tukey's multiple comparisons test | 5,5,5,5 | P<0.0001 | F (3, 16) = 34.59 |
| 5G | One-way ANOVA with Tukey's multiple comparisons test | 5,5,5,5 | P<0.0001 | F (3, 16) = 18.39 |
| 5J | One-way ANOVA with Tukey's multiple comparisons test | 16,16,16,16 | P<0.0001 | F (3, 60) = 23.94 |
| 5K | One-way ANOVA with Tukey's multiple comparisons test | 50,50,50,50 | P<0.0001 | F (3, 196) = 32.66 |
| 5M | One-way ANOVA with Tukey's multiple comparisons test | 10,10,10,10 | P<0.0001 | F (3, 36) = 32.60 |
| 5N | One-way ANOVA with Tukey's multiple comparisons test | 50,50,50,50 | P<0.0001 | F (3, 196) = 8.787 |
| 5O | One-way ANOVA with Tukey's multiple comparisons test | 50,50,50,50 | P=0.9610 | F (3, 196) = 0.09810 |
| 5Q | One-way ANOVA with Tukey's multiple comparisons test | 5,5,5,5 | P<0.0001 | F (3, 16) = 14.99 |
| 5R | One-way ANOVA with Tukey's multiple comparisons test | 9,9,9,9 | P=0.0001 | F (3, 32) = 9.650 |
| 5S | One-way ANOVA with Tukey's multiple comparisons test | 9,9,9,9 | P<0.0001 | F (3, 32) = 10.87 |
| 5T | Two-way ANOVA with Tukey's multiple comparisons test | 10,10,10,10 | P=0.1044  P<0.0001  P<0.0001 | F (12, 180) = 1.568  F (4, 180) = 53.72  F (3, 180) = 14.56 |
| 5U | One-way ANOVA with Tukey's multiple comparisons test | 10,10,10,10 | P=0.0015 | F (3, 36) = 6.304 |
| 5V | One-way ANOVA with Tukey's multiple comparisons test | 10,10,10,10 | P=0.0017 | F (3, 36) = 6.164 |
| 6C | One-way ANOVA with Tukey's multiple comparisons test | 5,5,5,5 | P<0.0001 | F (3, 16) = 14.29 |
| 6D | One-way ANOVA with Tukey's multiple comparisons test | 5,5,5,5 | P<0.0001 | F (3, 16) = 17.38 |
| 6G | One-way ANOVA with Tukey's multiple comparisons test | 5,5,5 | P<0.0001 | F (2, 12) = 25.99 |
| 6H | One-way ANOVA with Tukey's multiple comparisons test | 5,5,5 | P<0.0001 | F (2, 12) = 39.36 |
| 6I | One-way ANOVA with Tukey's multiple comparisons test | 5,5,5 | P<0.0001 | F (2, 12) = 32.96 |
| 6L | One-way ANOVA with Tukey's multiple comparisons test | 16,16,16 | P<0.0001 | F (2, 45) = 17.37 |
| S1B | Two-tailed t test | 4,4 | P=0.0004 | t=7.036, df=6 |
| S2B | One-way ANOVA with Tukey's multiple comparisons test | 4,4,4 | P=0.0034 | F (2, 9) = 11.42 |
| S3B | Two-tailed t test | 5,5 | P=0.7047 | t=0.3928, df=8 |
| S3C | Two-tailed t test | 5,5 | P=0.4340 | t=0.8236, df=8 |
| S4A | One-way ANOVA with Tukey's multiple comparisons test | 10,10,10 | P=0.7656 | F (2, 27) = 0.2697 |
| S4B | One-way ANOVA with Tukey's multiple comparisons test | 10,10,10 | P=0.2170 | F (2, 27) = 1.618 |
| S.5B | One-way ANOVA with Tukey's multiple comparisons test | 5,5,5,5 | P=0.1401 | F (3, 16) = 2.103 |
| S5C | One-way ANOVA with Tukey's multiple comparisons test | 5,5,5,5 | P=0.0324 | F (3, 16) = 3.757 |
| S6C | One-way ANOVA with Tukey's multiple comparisons test | 16,16,16,16 | P<0.0001 | F (3, 60) = 14.75 |
| S7A | One-way ANOVA with Tukey's multiple comparisons test | 10,10,10,10 | P=0.7175 | F (3, 36) = 0.4520 |
| S7B | One-way ANOVA with Tukey's multiple comparisons test | 10,10,10,10 | P=0.1298 | F (3, 36) = 2.011 |
| S8B | One-way ANOVA with Tukey's multiple comparisons test | 4,4,4,4 | P<0.0001 | F (3, 12) = 20.32 |
| S9C | One-way ANOVA with Tukey's multiple comparisons test | 15,15,15,15 | P<0.0001 | F (2, 42) = 13.77 |
| S9E | One-way ANOVA with Tukey's multiple comparisons test | 5, 5, 5, 5 | P<0.0001 | F (2, 12) = 24.55 |
| S9F | One-way ANOVA with Tukey's multiple comparisons test | 5, 5, 5, 5 | P=0.0004 | F (2, 12) = 16.27 |
| S9G | One-way ANOVA with Tukey's multiple comparisons test | 5, 5, 5, 5 | P=0.0002 | F (2, 12) = 18.30 |
